# PROTOCOL: Group‐based community interventions to support the social reintegration of marginalised adults with mental illness

**DOI:** 10.1002/cl2.1254

**Published:** 2022-07-14

**Authors:** Nina T. Dalgaard, Maya C. Flensborg Jensen, Elizabeth Bengtsen, Karl F. Krassel, Mikkel H. Vembye

**Affiliations:** ^1^ VIVE—The Danish Centre for Social Science Research Copenhagen Denmark

## Abstract

This is the protocol for a Campbell systematic review. The main objective is to explore the general efficacy of group‐based community interventions aimed at supporting marginalised adults with mental illness and related problems on outcomes such as problem behaviour, subjective well‐being, homelessness, poverty and employment. Furthermore, the objective is to explore the potential advantages/disadvantages of using a group‐based versus an individual intervention when targeting specific problems or when using specific types of interventions.

## 1 BACKGROUND

Adults suffering from mental illness constitute a vulnerable population with an increased risk of experiencing co‐morbidity. Common co‐morbid conditions include personal and social problems such as substance or alcohol abuse, self‐harming behaviour, criminal behaviour, homelessness, long‐term unemployment, poverty and social isolation. These problems increase the risk that mental illness leads to (social) marginalisation, stigmatisation and increased welfare costs (Draine et al., 2002; Lai et al., 2015; Nielsen et al., 2011; Schreiter et al., 2017).

Several studies suggest that mental illness, discrimination and (self‐) stigmatisation may become part of a vicious cycle. A cycle in which adults who suffer from mental illness abstain from engaging in social activities, which may lead to further marginalisation and sometimes to a further deterioration in mental health (Brouwers, 2020; Feldman & Crandall, 2007). For example, in a qualitative study based on interviews with 46 adults suffering from a wide range of mental health diagnoses, Dinos et al. (2004) found that participants described experiencing stigma even in the absence of overt discrimination by others or within society. In the study, participants describe how their experiences of stigma often cause stress, anxiety and rumination, and how this fear of being stigmatised leads to self‐isolating and self‐limiting behaviours. Many adults suffering from mental illness thereby have to cope with both their mental illness and their risk of social marginalisation at the same time.

To support the social reintegration of marginalised adults with mental illness and related problems, a number of interventions exist. For example, occupational therapy, intensive case management, psycho‐education, supportive psychotherapy or mentoring are targeting people with mental disorder and related problems (e.g., substance or alcohol abuse, criminal behaviour, homelessness and marginalisation). These interventions are costly and time consuming, and the evidence regarding their efficacy is far from unequivocal (Dutra et al., 2008; Sledge et al., 2011; Ziguras & Stuart, 2000). Therefore, more recently, the use of group‐based interventions has expanded as an alternative to individual therapy or other interventions.

### 1.1 Description of the condition

The growing demand for and use of group‐based interventions happen in a context where most high‐income countries' mental health services have been transformed from hospital‐centred to community‐based services. A transformation that leave more responsibility and/or the cost of treatment and interventions to community‐based services (Wahlbeck et al., 2011). From a community‐based service perspective the implementation of group‐based interventions is increasingly celebrated as a way to bridge the gap between a growing demand for treatment and limited budgets for outpatient interventions (Ruesch et al., 2015).

Group interventions have the advantage of being able to treat many patients simultaneously. Therefore, the costs are low (Ruesch et al., 2015). In addition, Ruesch et al. (2015) find that group‐based interventions in relation to depression treatment are marginally inferior or have similar effects as individual therapy. For patients with co‐morbid mental illness group‐based intervention may also be beneficial because the group offer social benefits through the reduction of the individual's feelings of loneliness and social isolation (Ruesch et al., 2015).

The high prevalence of personal and social co‐morbidities for psychiatric patients, the changed institutional setting in mental healthcare, and the popularity of group‐based community interventions (partly driven by budget concerns) create a demand for a thorough literature review in the field. Hence, the purpose of our review is to provide insights regarding efficacy of group‐based community interventions for marginalised adults with mental illness.

### 1.2 Description of the intervention

Group‐based interventions can be adapted for different (mental) disorders, age groups and diverse communities and settings. Group‐based interventions will often be provided in a small, selected group of individuals who meet regularly with a therapist or case worker (Fehr, 2019).

This review will include all interventions targeting adults who suffer from mental illness and related social and personal problems if the intervention is delivered in a group format, meaning that more than one participant receive the intervention at the same time and place and by the same therapists/case workers/mentors, etc. In addition, interventions must be based in a community or out‐patient setting. Furthermore, we will exclude psychiatric interventions based on psychopharmacological treatment alone and interventions taking place in hospital settings while patients are receiving around the clock care.

To be eligible for the present review, the group‐based intervention must be aimed at supporting the social reintegration of participants. This means that interventions with the sole focus of reducing symptoms of a specific mental health diagnosis will not be eligible. More specifically, the review will include all types of mental illness symptoms as long as the intervention also targets other aspects of the participants' lives and well‐being. Examples of personal/social problems, which the interventions may target are:

- Alcohol/substance abuse
- Self‐harming behaviour
- Criminal behaviour
- Homelessness
- Poverty
- Unemployment
- Hospital admissions
- Participants' subjective well‐being and quality of life
- Social isolation
- Feelings of loneliness

This list is not exhaustive, as we aim to define personal and social problems very broadly in order for the review to include all relevant studies.

Any adverse effects of interventions will be reported as an outcome.

### 1.3 How the intervention might work

Theoretically, group‐based interventions for adults suffering from mental illness aimed at supporting social reintegration may be understood through a *recovery* lens. The concept of recovery in mental health can be traced to the early 1980s, when personal accounts of individuals living with mental illness were published, describing their ability to live and cope with their mental illness (Gibson et al., 2011). As described by Anthony (1993), recovery is:

a deeply personal, unique process of changing one's attitudes, values, feelings, goals, skills, and/or roles. It is a way of living a satisfying, hopeful, and contributing life, even with limitations caused by the illness. Recovery involves the development of new meaning and purpose in one's life as one grows beyond the catastrophic effects of mental illness.

(Anthony, 1993 cited in Gibson et al. in p. 248)

Recovery can also be described as a process in which the individual may or may not experience a reduction in symptoms but in which the ability to cope with symptoms is improved enabling the individual to participate in social or occupational activities and to lead a meaningful life despite the mental illness. Thus, interventions, which will be included in the present review have a broader aim than to simply reduce the symptoms of mental illness. In essence, the aims are to help participants to form new relationships, develop coping and social skills enabling the participants to subsequently participate in more social and occupational contexts and to increase their general well‐being and quality of life. Theoretically group‐based interventions may also be seen through a *social identity* lens in which becoming members of a group may affect the social identity of marginalised individuals positively. According to Tarrant et al., 2012 health‐promoting behaviours are affected by social identity through the individual's adoption of norms of the group, and this may be seen as one of the central mechanisms of change in group‐based interventions.

#### 1.3.1 Advantages of group‐based interventions: Focus on interpersonal and (social) support factors

Socially marginalised adults suffering from mental illness constitute a highly diverse population with a multitude of challenges in terms of both mental and physical health. It is beyond the scope of the present review to present the specific risk and protective factors associated with each diagnosis, but what many of the diagnoses and conditions have in common is that interpersonal functioning and support constitute major predictive factors when studying relapse prevention and recurrence of symptoms following treatment (Brown & Lewinsohn, 1984; Hammen, 1991; Keitner & Miller, 1990). In addition, interpersonal and support factors are also one of the few changeable predictors in the course of illness (Keitner et al., 1992). This has high relevance for this review since, compared with individual therapy, the interpersonal and social support factor is an inherent part of group‐based interventions (Keitner et al., 1992; McDermut et al., 2001; Yalom, 1995). Thus, group interventions may address important factors in long‐term outcome of treatment of mental illness in ways that individual treatments may not, for example, individual's feelings of loneliness and social isolation (Ruesch et al., 2015). Thus, it can be suggested that group‐based interventions may add benefits to individual interventions, as the context of group processes are proposed to encourage social functioning and provide buffering effects of social support. Furthermore, previous studies suggest that when compared to individual interventions for psychiatric patients with bipolar disorder group‐based interventions may offer advantages in terms of self‐confidence, behaviour and social functioning but not on symptom reduction (Castle et al., 2007).

Furthermore, a study carried out by Colom and Vieta (2004) indicate that group‐based interventions offer advantages beyond the supportive effects of being placed in a group. Colom and Vieta (2004) compared a 21‐session group based psycho‐education intervention incorporating a number of key approaches of other interventions, including stress management techniques, problem‐solving, establishment of routines and strategies for managing warning signs with a befriending group (to control for the supportive effect of the group itself). The intervention group experienced a significant reduction in the number of participants who relapsed and number of recurrences per person. The number and length of hospitalisations were also lower for those in the intervention group.

#### 1.3.2 Deteriorating effects of (group‐)based interventions

The potential adverse effects of group psychotherapy or group interventions more broadly have not been the subject to the same scientific scrutiny as individual therapy (Roback Howard, 2000). However, the research into adverse outcomes and or *deterioration effects* in individual psychotherapy are well‐established and documented in several trials and systematic reviews. While we have argued that group and individual therapy are different types of treatment, they also share common characteristics. This makes the well‐established knowledge about the pitfalls of individual‐based therapy interesting from a group intervention perspective.

Based on Strupp Hans et al. (1977), the negative outcomes of individual psychotherapy that may occur during the course of treatment or following the end of treatment may include:

1. Exacerbation of presenting symptoms, for example, generalisation of symptoms.
2. Misuse/abuse of therapy, for example, patient substituting intellectualised insights for other obsessional thoughts.
3. Undertaking unrealistic goals or tasks, for example, pursuing goals that one is ill equipped to achieve in an attempt to please the therapist.
4. Loss of trust in therapy or the therapist, for example, patient's disillusionment prevents him or her from seeking out necessary therapy in the future.
5. Appearance of new symptoms (suicide would be an extreme example).

Regarding this last point, it should be noted, that it is often very difficult to determine if these negative outcomes were therapy‐induced or merely occurred at the time when the patient was receiving an ineffective treatment (Roback, 2000). In explaining these negative outcomes in individual psychotherapies, a number of studies document associations between characteristics of both therapist and patients and negative outcomes (e.g., some therapists appear be unsuitable or ineffective for patients with certain characteristics such as specific diagnoses, personality traits or underlying undiagnosed conditions). These effects are likely to be similar for group interventions (e.g., some patients and therapists are likely to be unfit for certain therapies when delivered in a group format). However, group interventions may also fail patients for reasons associated with the group. According to Roback Howard (2000):

A group is often more than the sum of its parts. At times, however, it may be less than the sum of its parts. Ideally, therapeutic groups develop a work culture under the skillful direction of a leader knowledgeable not only in the areas of psychopathology and psychodiagnostics, but also in group dynamics and interpersonal communication. That is, characteristics of the group itself become critical in treatment outcomes. Dynamic properties of therapeutic groups include factors such as intragroup cohesion, group norms, group roles, group pressure, conformity, communication structure, social comparison, and self‐disclosure.

(Roback, 2000; p. 117)

Theoretically, it is thus possible, that for some marginalised adults suffering from mental illness, group interventions may not bring about the expected positive change or they may even have negative effects. These potential negative effects may happen if the group lacks cohesion, if confidentiality is breached by participants in the group, or if participants feel rejected or invalidated by other participants during the intervention (Fehr, 2019). These negative characteristics or intra‐group dynamics may increase rather than decrease the participants' feeling of isolation, rejection and sense of self‐worth (Fehr, 2019). Thus, it is also possible that group interventions may be less effective than individual treatment for some.

In summary, group‐based interventions aimed at recovery and social reintegration of participants are proposed to offer advantages to patients when compared with both no treatment and with individual interventions in terms of psychosocial support, which is then proposed to lead to increased social and interpersonal functioning. The experience of social support and increased social and interpersonal functioning may subsequently constitute a prospective protective factor, and thus it is proposed that group‐based treatment may lead to more sustainable treatment results. However, previous research also points to the potential negative effects of group therapeutic interventions. Theoretically, it is possible that participants with certain characteristics (such as specific diagnoses, co‐morbidities or personality traits) will experience negative effects of group interventions and that for some participants individual interventions may be more effective.

### 1.4 Why it is important to do this review

A large body of reviews explore the efficacy of psychiatric group interventions targeting specific mental health disorders such as group psychotherapy for anxiety or personality disorders (Barkowski et al., 2020; McLaughlin et al., 2019; Burlingame et al., 2003). However, most reviews focus on symptom reduction as the only outcome, and are thus not relevant to the present review, in which we aim to explore the efficacy on a more broad range of outcomes associated with social reintegration and not just symptom reduction, for example, experience of a meaningful and social life *despite* the mental illness.

For the purpose of this review, we have identified six existing reviews, which include outcomes other than symptom reduction. The first two reviews that we present focus on the effects of outpatient psychiatric group interventions for a specific mental health diagnosis (psychosis and post‐traumatic stress disorder). In contrast, the remaining four reviews focuses on treatment for respectively illicit drug dependence, homelessness, substance abuse disorder and alcohol use disorder, which are examples of central comorbidities, which are often experienced by adults suffering from mental illness.

In a review on the effects of group programs for recovery from psychosis, Segredou et al. (2008) identified 20 studies, and concluded that findings suggest positive effects on participants' social and vocational functioning in addition to symptom reduction. However, they also conclude, that findings are uncertain, as many studies lack appropriate control groups, follow‐up and standardised measures of symptoms and diagnosis. The review which was presented as a conference poster provides a very limited description of the search process, no risk of bias assessment of included studies and they do not conduct a meta‐analysis.

Bøg et al. (2017) conducted a systematic review and meta‐analysis on the effectiveness of 12‐step interventions for participants with illicit drug dependence based on 10 randomised controlled trials and quasi‐experimental studies (*N* = 1071). In addition to the primary outcome of drug use the review included outcomes such as criminal behaviour, prostitution, psychiatric symptoms, social functioning, employment status and homelessness. The review concludes that there is no difference in the effectiveness of 12‐step interventions compared to alternative psychosocial interventions in reducing drug use during treatment, post treatment, and at 6‐ and 12‐month follow‐ups, furthermore the review found no statistically significant differences between 12‐step and another psychosocial interventions post‐treatment on measures of psychiatric symptoms, social functioning, and employment.

Munthe‐Kaas et al. (2018) conducted a systematic review and meta‐analysis on the effectiveness of interventions to reduce homelessness based on 43 samples. The review concludes that the included interventions; high‐intensity case management, housing first, critical time intervention (CTI), abstinence‐contingent housing, non‐abstinence‐contingent housing with high‐intensity case management, housing vouchers and residential treatment perform better than the usual services at reducing homelessness or improving housing stability in all comparisons. Furthermore it was concluded that group living arrangements may be better than individual apartments at reducing homelessness (low certainty evidence).

Mahoney et al. (2019) conducted a systematic review and meta‐analysis on the effects of group treatments for adults with symptoms associated with complex posttraumatic stress disorder based on 36 randomised controlled trials. Outcomes included four types of symptoms and substance misuse. Medium to large significant effect sizes favouring group‐based trauma interventions were found for four of the outcome domains with only substance misuse resulting in a small nonsignificant effect size.

In a systematic review and meta‐analysis on the effectiveness of group treatment for substance use disorder in adults based on 33 randomised clinical trials (*N* = 3951), Coco et al. (2019) compared group psychotherapy to no treatment control groups, individual psychotherapy, medication, self‐help groups, and other active treatments applying no specific psychotherapeutic techniques for patients with substance use disorder. The primary outcome was abstinence, and the secondary outcomes were frequency of substance use and symptoms of substance use disorder, anxiety, depression, general psychopathology, and attrition. Significant small effects of group therapy were found on abstinence compared to no treatment, individual therapy, and other treatments. Effects on substance use frequency and symptoms of substance use disorder were not significant, but significant moderately sized effects emerged for mental state when group therapy was compared to no treatment. There were no differences in abstinence rates between group therapy and control groups (Coco et al., 2019).

Group‐based interventions targeting comorbidities relevant for our population of interest have proven to be effective in general populations. A noticeable and recent example is a Cochrane review (Kelly et al., 2020) on the effect of Alcohol Anonymous (AA) and other 12‐step programs against alcohol use disorder (AUD). In its original form, AA works through a social fellowship (meetings with peers) and a 12‐step program. Hence, AA is considered group intervention/therapy. Kelly et al. (2020) review 27 studies (*N* = 10 565) and compare AA with motivational enhancement therapy (MET), cognitive behavioural therapy (CBT), variants of 12‐step programs and no treatment. Outcomes consists of a range of drinking‐related outcomes (abstinence, intensity, consequences and addiction severity) and healthcare cost offsets. Kelly et al. (2020) report evidence that AA results in longer periods of abstinence and AA perform as good as other treatments with respect to intensity, consequences and addiction severity. In addition, Kelly et al. (2020) report that four out of five studies found cost saving benefits, which in turn probably leads to reduced healthcare costs.

Our review adds to the existing body of reviews by exploring the efficacy of group interventions on a more broad range of outcomes, than what is seen in the existing reviews. Second, we will review interventions targeting a larger population (e.g., adults suffering from any kind of mental illness) and we will in include both community‐based and outpatient psychiatric interventions. Finally, we will provide a thorough risk of bias assessment of the included studies and if possible conduct meta‐analyses on outcomes, which are not included in the existing reviews.

The number of people with mental illness is growing in the Western world and both direct and indirect costs are expected to rise (Bloom et al., 2011). This growth force policymakers to reconsider how they can meet the increasing demand. Especially local governments, since psychiatric institutional care (hospital beds), is increasingly being replaced by out‐patient care (Wahlbeck et al., 2011).

The effects of psychiatric interventions aimed at reducing symptoms for patients with specific diagnoses have been extensively explored in a large number of reviews and meta‐analyses, but only a much smaller number of existing reviews have explored the effects of interventions on a broader range of measures. The present review will contribute to the knowledge base by including a broader range of outcomes: alcohol/substance abuse, self‐harming behaviour, criminal behaviour, homelessness, poverty, unemployment, hospital admissions, participants' subjective well‐being and quality of life.

As pointed out by McDaid and Park (2015) the economic cost of comorbidities have been remarkably neglected by health economists in health in general but also across mental and physical health. The relative increase in costs for comorbid diabetes is for example in the range of 1.8–2.0 for patients diagnosed with schizophrenia or depression. In addition, McDaid and Park (2015) point out that the costs of non‐health‐related comorbid conditions have been even more neglected despite clear evidence of much higher prevalence of non‐health‐related comorbidities among physical and mental health patients. As example, McDaid and Park (2015) points out that patients with major depressive disorder in Australian data have been found to have higher adjusted odds of 4.0 in difficulty of day to day work and higher adjusted odds of 1.7 in number of days unable to work. This underline the importance of considering a broader range of outcomes when assessing costs of mental health disorders (and health in general). A further underlining of this, is the finding by Stant et al. (2007) where group differences in the treatment of schizophrenia only revealed itself when using multiple health outcomes including the preference‐based QALY (Quality‐Adjusted Life Years) leading the authors to issue a caution when assessing the results of economic studies only using a single and specific outcome.

As previous noted, the cost of group‐based interventions can be less than half the cost of individual therapy (Ruesch et al., 2015). Yet, when policymakers choose group‐based community interventions they do so without having a solid knowledge base. Knowledge about the efficacy of group‐based community interventions in general, and when compared to individually delivered interventions, is thus crucial for policy makers in charge of deciding which interventions to fund.

## 2 OBJECTIVES

The main objective is to explore the general efficacy of group‐based community interventions aimed at supporting marginalised adults with mental illness and related problems on outcomes such as problem behaviour, subjective well‐being, homelessness, poverty and employment.

Furthermore, the objective is to explore the potential advantages/disadvantages of using a group‐based versus an individual intervention when targeting specific problems or when using specific types of interventions.

## 3 METHODS

### 3.1 Criteria for considering studies for this review

#### 3.1.1 Types of studies

Randomised controlled trials will be included. To summarise what is known about the possible causal effects of group‐based community interventions, we will include all study designs that use a well‐defined control group. Non‐randomised studies, where participants are assigned to conditions outside the researcher's control, must demonstrate pre‐treatment group equivalence via matching, statistical controls, or evidence of equivalence on key risk variables and participant characteristics. These factors are outlined in the section Assessment of risk of bias in included studies, and the methodological appropriateness of the included studies will be assessed according to the risk of bias.

The study designs we will include in the review are:

1) Randomised controlled trials (RCTs)
2) Quasi‐randomised controlled trial designs (QRCTs). Here participants are allocated by means, which are not expected to influence outcomes, for example, alternate allocation, participant's birth data, case number, or alphabetic order.
3) Quasi‐experimental studies (QES). This category refers to both studies, where participants are allocated by other actions controlled by the researcher, or where allocation to the intervention and control group are not controlled by the researcher (e.g., allocation according to time differences or policy rules).
4) Non‐randomised studies where there is a comparison of two or more groups of participants including studies comparing two different therapeutic modalities (i.e., without a control group)

Studies using single group pre–post comparisons will not be included.

#### 3.1.2 Types of participants

The population of this review are adults in the OECD countries with at least one psychiatric diagnosis who are experiencing any kind of personal and social problems in addition to their mental health problems. We will include participants with any kind of psychiatric diagnosis and we will include both studies in which patients self‐report on diagnosis and studies in which diagnosis are based on an assessment by a mental health professional. Social or personal problems is defined broadly and may include one or more of the following:

- Alcohol/substance abuse
- Self‐harming behaviour
- Criminal behaviour
- Homelessness
- Poverty
- Unemployment
- Hospital admissions
- Participants' subjective well‐being and quality of life
- Social isolation
- Feelings of loneliness

We will exclude studies of interventions targeting youth under the age of 18. Psychiatric patients, without any co‐morbid personal and social problems who receive out‐patient treatment for their specific mental disorder with symptom reduction as the primary aim will thus not be eligible.

#### 3.1.3 Types of interventions

This review will include all interventions targeting adults who suffer from mental illness and related social and personal problems if the intervention is delivered in a group format, meaning that more than one participant receive the intervention at the same time and place and by the same therapists/case workers/mentors etc. In addition, interventions must be based in a community or out‐patient setting as outlined in the section entitled: ‘The Intervention’. Comparison will include no treatment, treatment as usual/other interventions/treatments offered (including normal service provision) or waiting list control.

#### 3.1.4 Types of outcome measures

The relevant outcomes for the present review are in broader terms related to problem behaviours and social problems associated with social marginalisation. Included outcomes thus include, but are not limited to:

- Alcohol/substance abuse
- Self‐harming behaviour
- Criminal behaviour
- Homelessness
- Poverty
- Unemployment
- Hospital admissions
- Participants' subjective well‐being and quality of life

Any adverse effects of interventions will be reported as an outcome.

### 3.2 Primary outcomes

Based on the exploratory objectives for the present review, we do not distinguish between primary and secondary outcomes nor do we restrict ourselves to specific standardised outcome measures.

### 3.3 Secondary outcomes

#### 3.3.1 Duration of follow‐up

Time points for measures considered will be:

- 0–1 year follow‐up
- 1–2 years follow‐up
- >2 years follow‐up

Follow‐up at any given point in time will be included if meaningful based on the objectives for the review. This means that if possible, we will include follow‐up data reporting on the included outcomes during the remainder of the participants' life course.

#### 3.3.2 Types of settings

To be eligible for the present review, interventions must be based in a community or out‐patient setting and must be aimed at supporting the social reintegration of participants.

We will exclude interventions taking place in hospital settings while patients are receiving around the clock care. However, if patients are admitted to in‐hospital treatment and subsequently receive out‐patient group‐based services or interventions in a psychiatric or hospital setting this may also be included in the review

### 3.4 Search methods for identification of studies

To maximise coverage of the field of study while simultaneously attempting to reduce different types of bias, we implemented a range of search methods and strategies. The different strategies and methods will be presented below. ly describe the anticipated search strategy.

#### 3.4.1 Electronic searches

##### 3.4.1.1 Bibliographical databases

MEDLINE (OVID) 1966–2022
EMBASE (OVID) 1974–2022
APA PsycINFO (EBSCO) 1800–2022
CINAHL (EBSCO) 1981–2022
Sociological Abstracts (ProQuest) 1952–2022
Social Services Abstracts (ProQuest) 1979–2022
SocINDEX (EBSCO) 1908–2022
Academic Search Premier (EBSCO) 1975–2022
International Bibliography of the Social Sciences (IBSS) (ProQuest) 1951–2022
Science Citation Index (Web of Science Core Collection) 1900–2022
Social Sciences Citation Index (Web of Science Core Collection) 1990–2022
Cochrane Central Register of Controlled Trials (CENTRAL) (1996)–2022

##### 3.4.1.2 Example of search strings

Example of search strategy in a database with a thesaurus

APA PsycINFO (*1800–2022)*

Searched 22/05/2022

**Table:** 

#	Query	Expanders/Expanders	Results
S44	S38 AND S42	Expanders ‐ Apply equivalent subjects	4265
		Narrow by SubjectAge: ‐ very old (85 years and older)	
		Narrow by SubjectAge: ‐ aged (65 years and older)	
		Narrow by SubjectAge: ‐ thirties (30–39 years)	
		Narrow by SubjectAge: ‐ middle age (40–64 years)	
		Narrow by SubjectAge: ‐ young adulthood (18–29 years)	
		Narrow by SubjectAge: ‐ adulthood (18 years and older)	
		Search modes ‐ Boolean/Phrase	
S43	S38 AND S42	Expanders ‐ Apply equivalent subjects	6397
		Search modes ‐ Boolean/Phrase	
S42	S39 OR S40 OR S41	Expanders ‐ Apply equivalent subjects	2,169,833
		Search modes ‐ Boolean/Phrase	
S41	AB (((control* OR random* OR cluster‐random*) N3 (study OR studies OR group* OR trial* OR test* OR analy*)) OR effect* OR efficacy OR experiment* OR intervention* OR ‘exogenous variation’ OR ‘difference in difference’ OR ‘within household difference*’ OR ‘Regression discontinuity design*’ OR ‘RDD’ OR ‘RD’)	Expanders ‐ Apply equivalent subjects	1,971,577
		Search modes ‐ Boolean/Phrase	
S40	TI (((control* OR random* OR cluster‐random*) N3 (study OR studies OR group* OR trial* OR test* OR analy*)) OR effect* OR efficacy OR experiment* OR intervention* OR treatment* OR ‘exogenous variation’ OR ‘difference in difference’ OR ‘within household difference*’ OR ‘Regression discontinuity design*’ OR ‘RDD’ OR ‘RD’)	Expanders ‐ Apply equivalent subjects	676,685
		Search modes ‐ Boolean/Phrase	
S39	DE ‘Effect Size (Statistical)’ OR DE ‘Between Groups Design’ OR DE ‘Experimental Design’ OR DE ‘Clinical Trials’ OR DE ‘Intervention’ OR DE ‘Randomized Controlled Trials’ OR DE ‘Randomized Clinical Trials’ OR DE ‘Treatment Effectiveness Evaluation’ OR DE ‘Treatment Process and Outcome Measures’	Expanders ‐ Apply equivalent subjects	126,155
		Search modes ‐ Boolean/Phrase	
S38	S21 AND S28 AND S33 AND S37	Expanders ‐ Apply equivalent subjects	9232
		Search modes ‐ Boolean/Phrase	
S37	S34 OR S35 OR S36	Expanders ‐ Apply equivalent subjects	1,109,138
		Search modes ‐ Boolean/Phrase	
S36	(outpatient* OR out‐patient* OR discharge OR community OR communities OR outreach* OR ‘reach out’ OR ((health care OR healthcare OR mental OR treatment OR rehabilitation OR rehab) N3 (center* OR centre* OR facilit* OR service* OR site OR sites)) OR ‘alcoholics anonymous’ OR ‘social group*’ OR ‘support group*’)	Expanders ‐ Apply equivalent subjects	1,108,656
		Search modes ‐ Boolean/Phrase	
S35	DE ‘Outpatient Treatment’ OR DE ‘Outpatient Commitment’ OR DE ‘Outpatients’ OR DE ‘Outreach Programs’ OR DE ‘Alcoholics Anonymous’ OR DE ‘Social Groups’ OR DE ‘Support groups’	Expanders ‐ Apply equivalent subjects	29,065
		Search modes ‐ Boolean/Phrase	
S34	DE ‘Community Counseling’ OR DE ‘Community Mental Health’ OR DE ‘Assertive Community Treatment’ OR DE ‘Community Mental Health Centers’ OR DE ‘Community Mental Health Services’ OR DE ‘Community Mental Health Training’ OR DE ‘Mental Health Inservice Training’ OR DE ‘Community Services’ OR DE ‘Community Mental Health Services’ OR DE ‘Public Health Services’	Expanders ‐ Apply equivalent subjects	40,635
		Search modes ‐ Boolean/Phrase	
S33	S29 OR S30 OR S31 OR S32	Expanders ‐ Apply equivalent subjects	255,013
		Search modes ‐ Boolean/Phrase	
S32	SU (‘Group treatment*’ OR ‘group intervention’ OR ‘group counsel#ing’ OR ‘group therapy’ OR ‘group psychotherapy’ OR ‘group discussions’ OR ‘focus group*’ OR group‐based OR group‐oriented OR group‐focused OR group‐tailor* OR group‐centered OR group‐centred OR multi‐group*OR joint OR conjoint)	Expanders ‐ Apply equivalent subjects	45,902
		Search modes ‐ Boolean/Phrase	
S31	AB (Group N3 treatment*) OR (group N3 intervention) OR (group N3 counsel#ing) OR (group N3 therapy) OR (group N3 psychotherapy) OR (group N3 discussion*) OR ‘focus group*’ OR ‘client group*’ OR group‐based OR group‐oriented OR group‐focused OR group‐tailor* OR group‐centered OR group‐centred OR multi‐group* OR joint OR conjoint)	Expanders ‐ Apply equivalent subjects	178,640
		Search modes ‐ Boolean/Phrase	
S30	TI (Group* OR group‐based OR group‐oriented OR group‐focused OR group‐tailor* OR group‐centered OR group‐centred OR multi‐group* OR joint OR conjoint)	Expanders ‐ Apply equivalent subjects	95,311
		Search modes ‐ Boolean/Phrase	
S29	(((((DE ‘Group Counseling’ OR DE ‘Group Intervention’) OR (DE ‘Encounter Group Therapy’)) OR (DE ‘Therapeutic Community’)) OR (DE ‘Conjoint Therapy’ OR DE ‘Group Psychotherapy’)) OR (DE ‘Group Problem Solving’)) OR (DE ‘Alcoholics Anonymous’)	Expanders ‐ Apply equivalent subjects	35,933
		Search modes ‐ Boolean/Phrase	
S28	S22 OR S23 OR S24 OR S25 OR S26 OR S27	Expanders ‐ Apply equivalent subjects	789,406
		Search modes ‐ Boolean/Phrase	
S27	AB (Vulnerability OR Marginalization OR Stigmatization OR vulnerable OR multivulnerability OR multi‐vulnerability OR marginalised OR marginalized OR stigmatised OR stigmatized OR disadvantaged OR impoverished OR exposed OR underprivileged OR unprivileged OR underserved OR under‐served OR deprived OR ‘social problems’ OR ‘social exclusion’ OR ‘social excluded’ OR loneliness OR ‘drug misuse’ OR ‘drug abuse’ OR ‘drug dependent’ OR ‘drug dependency’ OR ‘substance disorder’ OR ‘substance disorders’ OR ‘substance dependency’ OR ‘substance dependent’ OR ‘substance abuse’ OR addiction OR homebound OR ‘multiple diagnoses’ OR ‘multiple illnesses’ OR ‘multiple chronic conditions’ OR comorbidity OR frail OR ‘functional loss’ OR ‘functional impairment’ OR ‘loss of function’ OR ‘functional disability’ OR ‘loss of adl’ OR homeless* OR houseless* OR shelter* OR ‘mentally impaired’ OR handicapped OR disability OR disabilities OR disabled OR ‘hard to reach’ OR poverty OR alcoholic* OR ‘alcohol abuse’ OR ‘alcohol misuse’ OR ‘alcohol problem’ OR ‘drinking problem’ OR ‘drug addiction’ OR ‘drug addict’ OR ‘drug addicts’ OR ‘drug user’ OR ‘drug users’ OR ‘drug use’ OR ‘drug misuse’ OR unemployed OR unemployment OR ‘health risk behavior’ OR ‘health risk behaviour’ OR ‘risky health behavior’ OR ‘risky health behaviour’ OR ‘risky life style’ OR ‘risky life‐style’ OR ‘risky lifestyle’ OR ‘low income’ OR ‘low in‐come’ OR ‘limited funds’ OR criminal* OR parolee* OR probation)	Expanders ‐ Apply equivalent subjects	605,582
		Search modes ‐ Boolean/Phrase	
S26	TI (Vulnerability OR Marginalization OR Stigmatization OR vulnerable OR multivulnerability OR multi‐vulnerability OR marginalised OR marginalized OR stigmatised OR stigmatized OR disadvantaged OR impoverished OR exposed OR underprivileged OR unprivileged OR underserved OR under‐served OR deprived OR ‘social problems’ OR ‘social exclusion’ OR ‘social excluded’ OR loneliness OR ‘drug misuse’ OR ‘drug abuse’ OR ‘drug dependent’ OR ‘drug dependency’ OR ‘substance disorder’ OR ‘substance disorders’ OR ‘substance dependency’ OR ‘substance dependent’ OR ‘substance abuse’ OR addiction OR homebound OR ‘multiple diagnoses’ OR ‘multiple illnesses’ OR ‘multiple chronic conditions’ OR comorbidity OR frail OR ‘functional loss’ OR ‘functional impairment’ OR ‘loss of function’ OR ‘functional disability’ OR ‘loss of adl’ OR homeless* OR houseless* OR shelter* OR ‘mentally impaired’ OR handicapped OR disability OR disabilities OR disabled OR ‘hard to reach’ OR poverty OR alcoholic* OR ‘alcohol abuse’ OR ‘alcohol misuse’ OR ‘alcohol problem’ OR ‘drinking problem’ OR ‘drug addiction’ OR ‘drug addict’ OR ‘drug addicts’ OR ‘drug user’ OR ‘drug users’ OR ‘drug use’ OR ‘drug misuse’ OR unemployed OR unemployment OR ‘health risk behavior’ OR ‘health risk behaviour’ OR ‘risky health behavior’ OR ‘risky health behaviour’ OR ‘risky life style’ OR ‘risky life‐style’ OR ‘risky lifestyle’ OR ‘low income’ OR ‘low in‐come’ OR ‘limited funds’ OR criminal* OR parolee* OR probation)	Expanders ‐ Apply equivalent subjects	178,814
		Search modes ‐ Boolean/Phrase	
S25	SU (Vulnerability OR Marginalization OR Stigmatization OR vulnerable OR multivulnerability OR multi‐vulnerability OR marginalised OR marginalized OR stigmatised OR stigmatized OR disadvantaged OR impoverished OR exposed OR underprivileged OR unprivileged OR underserved OR under‐served OR deprived OR ‘social problems’ OR ‘social exclusion’ OR ‘social excluded’ OR loneliness OR ‘drug misuse’ OR ‘drug abuse’ OR ‘drug dependent’ OR ‘drug dependency’ OR ‘substance disorder’ OR ‘substance disorders’ OR ‘substance dependency’ OR ‘substance dependent’ OR ‘substance abuse’ OR addiction OR homebound OR ‘multiple diagnoses’ OR ‘multiple illnesses’ OR ‘multiple chronic conditions’ OR comorbidity OR frail OR ‘functional loss’ OR ‘functional impairment’ OR ‘loss of function’ OR ‘functional disability’ OR ‘loss of adl’ OR homeless* OR houseless* OR shelter* OR ‘mentally impaired’ OR handicapped OR disability OR disabilities OR disabled OR ‘hard to reach’ OR poverty OR alcoholic* OR ‘alcohol abuse’ OR ‘alcohol misuse’ OR ‘alcohol problem’ OR ‘drinking problem’ OR ‘drug addiction’ OR ‘drug addict’ OR ‘drug addicts’ OR ‘drug user’ OR ‘drug users’ OR ‘drug use’ OR ‘drug misuse’ OR unemployed OR unemployment OR ‘health risk behavior’ OR ‘health risk behaviour’ OR ‘risky health behavior’ OR ‘risky health behaviour’ OR ‘risky life style’ OR ‘risky life‐style’ OR ‘risky lifestyle’ OR ‘low income’ OR ‘low in‐come’ OR ‘limited funds’ OR criminal* OR parolee* OR probation)	Expanders ‐ Apply equivalent subjects	435,518
		Search modes ‐ Boolean/Phrase	
S24	(((((DE ‘Homeless’ OR DE ‘Homeless Mentally Ill’ OR DE ‘Poverty’) OR (DE ‘Social Disadvantage’ OR DE ‘Unemployment’)) OR (DE ‘Poverty Reduction’ OR DE ‘Disadvantaged’)) OR (DE ‘Shelters’ OR DE ‘Social Deprivation’ OR DE ‘Social Isolation’)) OR (DE ‘Marginalized Groups’ OR DE ‘Minority Stress’ OR DE ‘Social Exclusion’)) OR (DE ‘Loneliness’)	Expanders ‐ Apply equivalent subjects	60,074
		Search modes ‐ Boolean/Phrase	
S23	DE ‘Substance Use Disorder’ OR DE ‘Addiction’ OR DE ‘Alcohol Use Disorder’ OR DE ‘Cannabis Use Disorder’ OR DE ‘Drug Abuse’ OR DE ‘Drug Dependency’ OR DE ‘Inhalant Abuse’ OR DE ‘Opioid Use Disorder’ OR DE ‘Drug Addiction’	Expanders ‐ Apply equivalent subjects	88,681
		Search modes ‐ Boolean/Phrase	
S22	((DE ‘Comorbidity’) OR (DE ‘Alcohol Intoxication’ OR DE ‘Alcohol Abuse’ OR DE ‘Alcoholism’)) OR (DE ‘Sobriety’)	Expanders ‐ Apply equivalent subjects	112,941
		Search modes ‐ Boolean/Phrase	
S21	S1 OR S2 OR S3 OR S4 OR S5 OR S6 OR S7 OR S8 OR S9 OR S10 OR S11 OR S12 OR S13 OR S14 OR S15 OR S16 OR S17 OR S18 OR S19 OR S20	Expanders ‐ Apply equivalent subjects	1,068,025
		Search modes ‐ Boolean/Phrase	
S20	AB (Mental disorder* OR mental disease* OR Mental illness* OR mental health OR Psychiatric diagnose OR Psychiatric illness* OR Psychiatric disease*OR Anxiety disorder* OR Phobic disorder* OR Social Anxiety OR ‘Generalized Anxiety Disorder’ OR ‘Obsessive Compulsive Disorder’ OR ‘Panic Attack’ OR ‘Panic Disorder’ OR ‘Acrophobia’ OR ‘Agoraphobia’ OR ‘Claustrophobia’ OR ‘Social Phobia’ OR OCD OR Antisocial disorder* OR Dissociative Disorder* OR Attention Deficit Disorder OR ADHD OR Hyperkinetic disorder* OR Asperger* OR Mood disorder* OR borderline OR Neurotic disorder* OR Personality disorder* OR Depression OR Bipolar disorder OR Schizophrenia OR Posttraumatic Stress Disorder OR Post‐traumatic Stress Disorder OR PTSD OR paranoia OR Psychosis OR Incest OR DE Pedophil* OR Voyeurism OR Eating Disorder* OR Anorexia OR Binge Eating OR Bulimia OR Narcissism OR Self‐Destructive Behavio#r OR Self‐Injurious Behavio#r OR Dissociative Disorder* OR Depersonalization OR Psychotic State OR borderline OR borderliner* OR neurosis OR Personality Disorder* OR Antisocial Personality Disorder OR Avoidant Personality Disorder OR Dependent Personality Disorder OR Narcissistic Personality Disorder OR Obsessive Compulsive Personality Disorder OR Paranoid Personality Disorder OR Passive Aggressive Personality Disorder OR Sadomasochistic Personality OR Schizoid Personality Disorder OR Schizophrenia OR bipolar disorder OR mania OR Autism OR autistic OR Affective Disorder* OR Disruptive Mood Dysregulation Disorder* OR mood disorder OR Major Depression OR Depression OR Endogenous Depression’ OR ‘Affective Psychosis’ OR ‘Schizoaffective Disorder’ OR Sadism OR Masochistic Personality)	Expanders ‐ Apply equivalent subjects	824,076
		Search modes ‐ Boolean/Phrase	
S19	TI (Mental disorder* OR mental disease* OR Mental illness* OR mental health OR Psychiatric diagnose OR Psychiatric illness* OR Psychiatric disease*OR Anxiety disorder* OR Phobic disorder* OR Social Anxiety OR ‘Generalized Anxiety Disorder’ OR ‘Obsessive Compulsive Disorder’ OR ‘Panic Attack’ OR ‘Panic Disorder’ OR ‘Acrophobia’ OR ‘Agoraphobia’ OR ‘Claustrophobia’ OR ‘Social Phobia’ OR OCD OR Antisocial disorder* OR Dissociative Disorder* OR Attention Deficit Disorder OR ADHD OR Hyperkinetic disorder* OR Asperger* OR Mood disorder* OR borderline OR Neurotic disorder* OR Personality disorder* OR Depression OR Bipolar disorder OR Schizophrenia OR Posttraumatic Stress Disorder OR Post‐traumatic Stress Disorder OR PTSD OR paranoia OR Psychosis OR Incest OR DE Pedophil* OR Voyeurism OR Eating Disorder* OR Anorexia OR Binge Eating OR Bulimia OR Narcissism OR Self‐Destructive Behavio#r OR Self‐Injurious Behavio#r OR Dissociative Disorder* OR Depersonalization OR Psychotic State OR borderline OR borderliner* OR neurosis OR Personality Disorder* OR Antisocial Personality Disorder OR Avoidant Personality Disorder OR Dependent Personality Disorder OR Narcissistic Personality Disorder OR Obsessive Compulsive Personality Disorder OR Paranoid Personality Disorder OR Passive Aggressive Personality Disorder OR Sadomasochistic Personality OR Schizoid Personality Disorder OR Schizophrenia OR bipolar disorder OR mania OR Autism OR autistic OR Affective Disorder* OR Disruptive Mood Dysregulation Disorder* OR mood disorder OR Major Depression OR Depression OR Endogenous Depression’ OR ‘Affective Psychosis’ OR ‘Schizoaffective Disorder’ OR Sadism OR Masochistic Personality)	Expanders ‐ Apply equivalent subjects	426,981
		Search modes ‐ Boolean/Phrase	
S18	SU (Mental disorder* OR mental disease* OR Mental illness* OR mental health OR Psychiatric diagnose OR Psychiatric illness* OR Psychiatric disease*OR Anxiety disorder* OR Phobic disorder* OR Social Anxiety OR ‘Generalized Anxiety Disorder’ OR ‘Obsessive Compulsive Disorder’ OR ‘Panic Attack’ OR ‘Panic Disorder’ OR ‘Acrophobia’ OR ‘Agoraphobia’ OR ‘Claustrophobia’ OR ‘Social Phobia’ OR OCD OR Antisocial disorder* OR Dissociative Disorder* OR Attention Deficit Disorder OR ADHD OR Hyperkinetic disorder* OR Asperger* OR Mood disorder* OR borderline OR Neurotic disorder* OR Personality disorder* OR Depression OR Bipolar disorder OR Schizophrenia OR Posttraumatic Stress Disorder OR Post‐traumatic Stress Disorder OR PTSD OR paranoia OR Psychosis OR Incest OR DE Pedophil* OR Voyeurism OR Eating Disorder* OR Anorexia OR Binge Eating OR Bulimia OR Narcissism OR Self‐Destructive Behavior OR Self‐Injurious Behavio#r OR Self‐Injurious Behavio#r OR Dissociative Disorder* OR Depersonalization OR Psychotic State OR borderline OR borderliner* OR neurosis OR Personality Disorder* OR Antisocial Personality Disorder OR Avoidant Personality Disorder OR Dependent Personality Disorder OR Narcissistic Personality Disorder OR Obsessive Compulsive Personality Disorder OR Paranoid Personality Disorder OR Passive Aggressive Personality Disorder OR Sadomasochistic Personality OR Schizoid Personality Disorder OR Schizophrenia OR bipolar disorder OR mania OR Autism OR autistic OR Affective Disorder* OR Disruptive Mood Dysregulation Disorder* OR mood disorder OR Major Depression OR Depression OR Endogenous Depression’ OR ‘Affective Psychosis’ OR ‘Schizoaffective Disorder’ OR Sadism OR Masochistic Personality)	Expanders ‐ Apply equivalent subjects	847,656
		Search modes ‐ Boolean/Phrase	
S17	DE ‘Thought Disturbances’ OR DE ‘Confabulation’ OR DE ‘Delusions’ OR DE ‘Fantasies (Thought Disturbances)’ OR DE ‘Fragmentation (Schizophrenia)’ OR DE ‘Judgment Disturbances’ OR DE ‘Magical Thinking’ OR DE ‘Memory Disorders’ OR DE ‘Obsessions’ OR DE ‘Perseveration’	Expanders ‐ Apply equivalent subjects	27,970
		Search modes ‐ Boolean/Phrase	
S16	DE ‘Stress and Trauma Related Disorders’ OR DE ‘Acute Stress Disorder’ OR DE ‘Adjustment Disorders’ OR DE ‘Attachment Disorders’ OR DE ‘Posttraumatic Stress Disorder’ OR DE ‘Posttraumatic Stress’	Expanders ‐ Apply equivalent subjects	41,703
		Search modes ‐ Boolean/Phrase	
S15	(DE ‘Hypersomnia’) OR (DE ‘Insomnia’)	Expanders ‐ Apply equivalent subjects	7758
		Search modes ‐ Boolean/Phrase	
S14	((DE ‘Acute Psychosis’ OR DE ‘Psychosis’) OR (DE ‘Affective Psychosis’ OR DE ‘Alcoholic Psychosis’)) OR (DE ‘Capgras Syndrome’ OR DE ‘Chronic Psychosis’ OR DE ‘Experimental Psychosis’ OR DE ‘Hallucinosis’ OR DE ‘Paranoia (Psychosis)’ OR DE ‘Postpartum Psychosis’ OR DE ‘Reactive Psychosis’ OR DE ‘Schizophrenia’ OR DE ‘Senile Psychosis’ OR DE ‘Toxic Psychoses’)	Expanders ‐ Apply equivalent subjects	127,378
		Search modes ‐ Boolean/Phrase	
S13	DE ‘Exhibitionism’ OR DE ‘Voyeurism’ OR DE ‘Fetishism’ OR DE ‘Incest’ OR DE ‘Pedophilia’ OR DE ‘Sadomasochism’ OR DE ‘Masochism’ OR DE ‘Sadism’	Expanders ‐ Apply equivalent subjects	7743
		Search modes ‐ Boolean/Phrase	
S12	(DE ‘Neuroticism’) OR (DE ‘Neurosis’ OR DE ‘Traumatic Neurosis’)	Expanders ‐ Apply equivalent subjects	13,362
		Search modes ‐ Boolean/Phrase	
S11	(((DE ‘Attention Deficit Disorder’ OR DE ‘Attention Deficit Disorder with Hyperactivity’ OR DE ‘Oppositional Defiant Disorder’) OR (DE ‘Disruptive Behavior Disorders’ OR DE ‘Conduct Disorder’)) OR (DE ‘Impulse Control Disorders’ OR DE ‘Behavior Disorders’ OR DE ‘Explosive Disorder’ OR DE ‘Pyromania’)) OR (DE ‘Self‐Destructive Behavior’ OR DE ‘Self‐Injurious Behavior’))	Expanders ‐ Apply equivalent subjects	52,409
		Search modes ‐ Boolean/Phrase	
S10	DE ‘Eating Disorders’ OR DE ‘Anorexia Nervosa’ OR DE ‘Binge Eating Disorder’ OR DE ‘Bulimia’ OR DE ‘Binge Eating’	Expanders ‐ Apply equivalent subjects	33,917
		Search modes ‐ Boolean/Phrase	
S9	DE ‘Dissociative Disorders’ OR DE ‘Depersonalization’ OR DE ‘Depersonalization/Derealization Disorder’ OR DE ‘Dissociative Amnesia’ OR DE ‘Dissociative Identity Disorder’ OR DE ‘Fugue Reaction’	Expanders ‐ Apply equivalent subjects	7,557
		Search modes ‐ Boolean/Phrase	
S8	DE ‘Chronic Mental Illness’ OR DE ‘Chronic Psychosis’	Expanders ‐ Apply equivalent subjects	1985
		Search modes ‐ Boolean/Phrase	
S7	(DE ‘Neurosis’ OR DE ‘Borderline States’) OR (DE ‘Psychosis’)	Expanders ‐ Apply equivalent subjects	40,838
		Search modes ‐ Boolean/Phrase	
S6	DE ‘Personality Disorders’ OR DE ‘Antisocial Personality Disorder’ OR DE ‘Avoidant Personality Disorder’ OR DE ‘Borderline Personality Disorder’ OR DE ‘Dependent Personality Disorder’ OR DE ‘Histrionic Personality Disorder’ OR DE ‘Narcissistic Personality Disorder’ OR DE ‘Obsessive Compulsive Personality Disorder’ OR DE ‘Paranoid Personality Disorder’ OR DE ‘Passive Aggressive Personality Disorder’ OR DE ‘Sadomasochistic Personality’ OR DE ‘Schizoid Personality Disorder’ OR DE ‘Schizotypal Personality Disorder’	Expanders ‐ Apply equivalent subjects	43,120
		Search modes ‐ Boolean/Phrase	
S5	DE ‘Bipolar Disorder’ OR DE ‘Bipolar I Disorder’ OR DE ‘Bipolar II Disorder’ OR DE ‘Cyclothymic Disorder’ OR DE ‘Mania’	Expanders ‐ Apply equivalent subjects	39,283
		Search modes ‐ Boolean/Phrase	
S4	DE ‘Autism Spectrum Disorders’ OR DE ‘Autistic Traits’	Expanders ‐ Apply equivalent subjects	50,540
		Search modes ‐ Boolean/Phrase	
S3	(((((((((((((DE ‘Affective Disorders’) OR (DE ‘Disruptive Mood Dysregulation Disorder’)) OR (DE ‘Major Depression’)) OR (DE ‘Depression (Emotion)’)) OR (DE ‘Anaclitic Depression’)) OR (DE ‘Dysthymic Disorder’)) OR (DE ‘Endogenous Depression’)) OR (DE ‘Late Life Depression’)) OR (DE ‘Reactive Depression’)) OR (DE ‘Recurrent Depression’)) OR (DE ‘Treatment Resistant Depression’)) OR (DE ‘Seasonal Affective Disorder’)) OR (DE ‘Affective Psychosis’)) OR (DE ‘Schizoaffective Disorder’)	Expanders ‐ Apply equivalent subjects	184,698
		Search modes ‐ Boolean/Phrase	
S2	(DE ‘Obsessive Compulsive Disorder’ OR DE ‘Social Anxiety’ OR DE ‘Generalized Anxiety Disorder’ OR DE ‘Panic Attack’ OR DE ‘Panic Disorder’ OR DE ‘Acrophobia’ OR DE ‘Agoraphobia’ OR DE ‘Claustrophobia’ OR DE ‘Social Phobia’) OR (DE ‘Anxiety Disorders’)	Expanders ‐ Apply equivalent subjects	64,604
		Search modes ‐ Boolean/Phrase	
S1	DE ‘Mental Disorders’ OR DE ‘Serious Mental Illness’ OR DE ‘Homeless Mentally Ill’ OR DE ‘Mentally Ill Offenders’	Expanders ‐ Apply equivalent subjects	143,629
		Search modes ‐ Boolean/Phrase	

##### 3.4.1.3 Example of search strategy in a database without a thesaurus

SocINDEX (EBSCO) 1908–2022

Expanders: Apply equivalent subjects

Search modes: Boolean/Phrase

Searched 23/05/2022

**Table:** 

#	Query	Results
S43	S38 AND S42	1587
S42	S39 OR S40 OR S41	500,000
S41	AB (((control* OR random* OR cluster‐random*) N3 (study OR studies OR group* OR trial* OR test* OR analy*)) OR effect* OR efficacy OR experiment* OR intervention* OR ‘exogenous variation’ OR ‘difference in difference’ OR ‘within household difference*’ OR ‘Regression discontinuity design*’ OR ‘RDD’ OR ‘RD’)	454,986
S40	TI (((control* OR random* OR cluster‐random*) N3 (study OR studies OR group* OR trial* OR test* OR analy*)) OR effect* OR efficacy OR experiment* OR intervention* OR treatment* OR ‘exogenous variation’ OR ‘difference in difference’ OR ‘within household difference*’ OR ‘Regression discontinuity design*’ OR ‘RDD’ OR ‘RD’)	125,192
S39	(DE ‘CLINICAL trials’ OR DE ‘RANDOMIZED controlled trials’ OR DE ‘INTERVENTION (Social services)’) OR (DE ‘OUTCOME assessment (Social services)’)	8011
S38	S20 AND S28 AND S33 AND S37	2518
S37	S34 OR S35 OR S36	507,336
S36	(((((DE ‘OUTPATIENT medical care’) OR (DE ‘OUTREACH programs’)) OR (DE ‘OUTPATIENT mental health facilities’)) OR (DE ‘OUTPATIENT substance abuse treatment facilities’)) OR (DE ‘GROUP counseling’ OR DE ‘SUPPORT groups’)) OR (DE ‘SOCIAL group work’)	4918
S35	(DE ‘COMMUNITY mental health services’ OR DE ‘COMMUNITY health services’) OR (DE ‘PUBLIC health’)	30,025
S34	(outpatient* OR out‐patient* OR discharge OR community OR communities OR outreach* OR ‘reach out’ OR ((health care OR healthcare OR mental OR treatment OR rehabilitation OR rehab) N3 (center* OR centre* OR facilit* OR service* OR site OR sites)) OR ‘alcoholics anonymous’ OR ‘social group*’ OR ‘support group*’)	496,168
S33	S29 OR S30 OR S31 OR S32	75,477
S32	AB (Group N3 treatment*) OR (group N3 intervention) OR (group N3 counsel#ing) OR (group N3 therapy) OR (group N3 psychotherapy) OR (group N3 discussion*) OR ‘focus group*’ OR ‘client group*’ OR group‐based OR group‐oriented OR group‐focused OR group‐tailor* OR group‐centered OR group‐centred OR multi‐group* OR joint OR conjoint)	45,112
S31	TI Group* OR group‐based OR group‐oriented OR group‐focused OR group‐tailor* OR group‐centered OR group‐centred OR multi‐group* OR joint OR conjoint	34,816
S30	((DE ‘GROUP psychotherapy’) OR (DE ‘GROUP relations training’ OR DE ‘GROUP psychotherapy’)) OR (DE ‘CONJOINT therapy’ OR DE ‘GROUP problem solving’)	4851
S29	SU ‘Group treatment*’ OR ‘group intervention’ OR ‘group counsel#ing’ OR ‘group therapy’ OR ‘group psychotherapy’ OR ‘group discussions’ OR ‘focus group*’ OR group‐based OR group‐oriented OR group‐focused OR group‐tailor* OR group‐centered OR group‐centred OR multi‐group*OR joint OR conjoint	10,258
S28	S24 OR S27	417,541
S27	S25 OR S26	338,509
S26	AB Vulnerability OR Marginalization OR Stigmatization OR vulnerable OR multivulnerability OR multi‐vulnerability OR marginalised OR marginalized OR stigmatised OR stigmatized OR disadvantaged OR impoverished OR exposed OR underprivileged OR unprivileged OR underserved OR under‐served OR deprived OR ‘social problems’ OR ‘social exclusion’ OR ‘social excluded’ OR loneliness OR ‘drug misuse’ OR ‘drug abuse’ OR ‘drug dependent’ OR ‘drug dependency’ OR ‘substance disorder’ OR ‘substance disorders’ OR ‘substance dependency’ OR ‘substance dependent’ OR ‘substance abuse’ OR addiction OR homebound OR ‘multiple diagnoses’ OR ‘multiple illnesses’ OR ‘multiple chronic conditions’ OR comorbidity OR frail OR ‘functional loss’ OR ‘functional impairment’ OR ‘loss of function’ OR ‘functional disability’ OR ‘loss of adl’ OR homeless* OR houseless* OR shelter* OR ‘mentally impaired’ OR handicapped OR disability OR disabilities OR disabled OR ‘hard to reach’ OR poverty OR alcoholic* OR ‘alcohol abuse’ OR ‘alcohol misuse’ OR ‘alcohol problem’ OR ‘drinking problem’ OR ‘drug addiction’ OR ‘drug addict’ OR ‘drug addicts’ OR ‘drug user’ OR ‘drug users’ OR ‘drug use’ OR ‘drug misuse’ OR unemployed OR unemployment OR ‘health risk behavior’ OR ‘health risk behaviour’ OR ‘risky health behavior’ OR ‘risky health behaviour’ OR ‘risky life style’ OR ‘risky life‐style’ OR ‘risky lifestyle’ OR ‘low income’ OR ‘low in‐come’ OR ‘limited funds’ OR criminal* OR parolee* OR probation	312,614
S25	TI Vulnerability OR Marginalization OR Stigmatization OR vulnerable OR multivulnerability OR multi‐vulnerability OR marginalised OR marginalized OR stigmatised OR stigmatized OR disadvantaged OR impoverished OR exposed OR underprivileged OR unprivileged OR underserved OR under‐served OR deprived OR ‘social problems’ OR ‘social exclusion’ OR ‘social excluded’ OR loneliness OR ‘drug misuse’ OR ‘drug abuse’ OR ‘drug dependent’ OR ‘drug dependency’ OR ‘substance disorder’ OR ‘substance disorders’ OR ‘substance dependency’ OR ‘substance dependent’ OR ‘substance abuse’ OR addiction OR homebound OR ‘multiple diagnoses’ OR ‘multiple illnesses’ OR ‘multiple chronic conditions’ OR comorbidity OR frail OR ‘functional loss’ OR ‘functional impairment’ OR ‘loss of function’ OR ‘functional disability’ OR ‘loss of adl’ OR homeless* OR houseless* OR shelter* OR ‘mentally impaired’ OR handicapped OR disability OR disabilities OR disabled OR ‘hard to reach’ OR poverty OR alcoholic* OR ‘alcohol abuse’ OR ‘alcohol misuse’ OR ‘alcohol problem’ OR ‘drinking problem’ OR ‘drug addiction’ OR ‘drug addict’ OR ‘drug addicts’ OR ‘drug user’ OR ‘drug users’ OR ‘drug use’ OR ‘drug misuse’ OR unemployed OR unemployment OR ‘health risk behavior’ OR ‘health risk behaviour’ OR ‘risky health behavior’ OR ‘risky health behaviour’ OR ‘risky life style’ OR ‘risky life‐style’ OR ‘risky lifestyle’ OR ‘low income’ OR ‘low in‐come’ OR ‘limited funds’ OR criminal* OR parolee* OR probation	113,833
S24	S21 OR S22 OR S23	226,168
S23	((((DE ‘HOMELESS shelters’ OR DE ‘HOMELESSNESS’ OR DE ‘SQUATTERS’) OR (DE ‘POVERTY’)) OR (DE ‘OUTCASTS’ OR DE ‘SOCIAL marginality’ OR DE ‘UNEMPLOYMENT’)) OR (DE ‘SOCIAL isolation’ OR DE ‘LONELINESS’)) OR (DE ‘MINORITY stress’)	42,197
S22	((((DE ‘ALCOHOLISM’ OR DE ‘ALCOHOLIC intoxication’) OR (DE ‘SUBSTANCE abuse’)) OR (DE ‘ADDICTIONS’ OR DE ‘BINGE drinking’ OR DE ‘MARIJUANA abuse’ OR DE ‘DRUG abuse’)) OR (DE ‘DRUG addiction’)) OR (DE ‘INHALANT abuse’)	43,633
S21	SU Vulnerability OR Marginalization OR Stigmatization OR vulnerable OR multivulnerability OR multi‐vulnerability OR marginalised OR marginalized OR stigmatised OR stigmatized OR disadvantaged OR impoverished OR exposed OR underprivileged OR unprivileged OR underserved OR under‐served OR deprived OR ‘social problems’ OR ‘social exclusion’ OR ‘social excluded’ OR loneliness OR ‘drug misuse’ OR ‘drug abuse’ OR ‘drug dependent’ OR ‘drug dependency’ OR ‘substance disorder’ OR ‘substance disorders’ OR ‘substance dependency’ OR ‘substance dependent’ OR ‘substance abuse’ OR addiction OR homebound OR ‘multiple diagnoses’ OR ‘multiple illnesses’ OR ‘multiple chronic conditions’ OR comorbidity OR frail OR ‘functional loss’ OR ‘functional impairment’ OR ‘loss of function’ OR ‘functional disability’ OR ‘loss of adl’ OR homeless* OR houseless* OR shelter* OR ‘mentally impaired’ OR handicapped OR disability OR disabilities OR disabled OR ‘hard to reach’ OR poverty OR alcoholic* OR ‘alcohol abuse’ OR ‘alcohol misuse’ OR ‘alcohol problem’ OR ‘drinking problem’ OR ‘drug addiction’ OR ‘drug addict’ OR ‘drug addicts’ OR ‘drug user’ OR ‘drug users’ OR ‘drug use’ OR ‘drug misuse’ OR unemployed OR unemployment OR ‘health risk behavior’ OR ‘health risk behaviour’ OR ‘risky health behavior’ OR ‘risky health behaviour’ OR ‘risky life style’ OR ‘risky life‐style’ OR ‘risky lifestyle’ OR ‘low income’ OR ‘low in‐come’ OR ‘limited funds’ OR criminal* OR parolee* OR probation	210,654
S20	S16 OR S19	159,529
S19	S17 OR S18	158,043
S18	AB Mental disorder* OR mental disease* OR Mental illness* OR mental health OR Psychiatric diagnose OR Psychiatric illness* OR Psychiatric disease*OR Anxiety disorder* OR Phobic disorder* OR Social Anxiety OR ‘Generalized Anxiety Disorder’ OR ‘Obsessive Compulsive Disorder’ OR ‘Panic Attack’ OR ‘Panic Disorder’ OR ‘Acrophobia’ OR ‘Agoraphobia’ OR ‘Claustrophobia’ OR ‘Social Phobia’ OR OCD OR Antisocial disorder* OR Dissociative Disorder* OR Attention Deficit Disorder OR ADHD OR Hyperkinetic disorder* OR Asperger* OR Mood disorder* OR borderline OR Neurotic disorder* OR Personality disorder* OR Depression OR Bipolar disorder OR Schizophrenia OR Posttraumatic Stress Disorder OR Post‐traumatic Stress Disorder OR PTSD OR paranoia OR Psychosis OR Incest OR DE Pedophil* OR Voyeurism OR Eating Disorder* OR Anorexia OR Binge Eating OR Bulimia OR Narcissism OR Self‐Destructive Behavio#r OR Self‐Injurious Behavio#r OR Dissociative Disorder* OR Depersonalization OR Psychotic State OR borderline OR borderliner* OR neurosis OR Personality Disorder* OR Antisocial Personality Disorder OR Avoidant Personality Disorder OR Dependent Personality Disorder OR Narcissistic Personality Disorder OR Obsessive Compulsive Personality Disorder OR Paranoid Personality Disorder OR Passive Aggressive Personality Disorder OR Sadomasochistic Personality OR Schizoid Personality Disorder OR Schizophrenia OR bipolar disorder OR mania OR Autism OR autistic OR Affective Disorder* OR Disruptive Mood Dysregulation Disorder* OR mood disorder OR Major Depression OR Depression OR Endogenous Depression’ OR ‘Affective Psychosis’ OR ‘Schizoaffective Disorder’	157,960
S17	TI Mental disorder* OR mental disease* OR Mental illness* OR mental health OR Psychiatric diagnose OR Psychiatric illness* OR Psychiatric disease*OR Anxiety disorder* OR Phobic disorder* OR Social Anxiety OR ‘Generalized Anxiety Disorder’ OR ‘Obsessive Compulsive Disorder’ OR ‘Panic Attack’ OR ‘Panic Disorder’ OR ‘Acrophobia’ OR ‘Agoraphobia’ OR ‘Claustrophobia’ OR ‘Social Phobia’ OR OCD OR Antisocial disorder* OR Dissociative Disorder* OR Attention Deficit Disorder OR ADHD OR Hyperkinetic disorder* OR Asperger* OR Mood disorder* OR borderline OR Neurotic disorder* OR Personality disorder* OR Depression OR Bipolar disorder OR Schizophrenia OR Posttraumatic Stress Disorder OR Post‐traumatic Stress Disorder OR PTSD OR paranoia OR Psychosis OR Incest OR DE Pedophil* OR Voyeurism OR Eating Disorder* OR Anorexia OR Binge Eating OR Bulimia OR Narcissism OR Self‐Destructive Behavior OR Self‐Injurious Behavio#r OR Self‐Injurious Behavio#r OR Dissociative Disorder* OR Depersonalization OR Psychotic State OR borderline OR borderliner* OR neurosis OR Personality Disorder* OR Antisocial Personality Disorder OR Avoidant Personality Disorder OR Dependent Personality Disorder OR Narcissistic Personality Disorder OR Obsessive Compulsive Personality Disorder OR Paranoid Personality Disorder OR Passive Aggressive Personality Disorder OR Sadomasochistic Personality OR Schizoid Personality Disorder OR Schizophrenia OR bipolar disorder OR mania OR Autism OR autistic OR Affective Disorder* OR Disruptive Mood Dysregulation Disorder* OR mood disorder OR Major Depression OR Depression OR Endogenous Depression’ OR ‘Affective Psychosis’ OR ‘Schizoaffective Disorder’	157,275
S16	S1 OR S2 OR S3 OR S4 OR S5 OR S6 OR S7 OR S8 OR S9 OR S10 OR S11 OR S12 OR S13 OR S14 OR S15	158,724
S15	DE ‘Post‐traumatic stress disorder’ OR DE ‘Post‐traumatic stress’	6532
S14	DE ‘SLEEP deprivation ‐‐ Social aspects’	11
S13	(((DE ‘PSYCHOSES’ OR DE ‘SCHIZOPHRENIA’) OR (DE ‘PARANOIA’))) OR (DE ‘SENILE dementia’)	4310
S12	(DE ‘EXHIBITIONISM (Sexual behavior)’ OR DE ‘FETISHISM (Religion)’) OR (DE ‘INCEST’ OR DE ‘PEDOPHILIA’ OR DE ‘SADOMASOCHISM’ OR DE ‘MASOCHISM’ OR DE ‘SADISM’)	2216
S11	DE ‘NEUROTICISM’ OR DE ‘NEUROSES’	1363
S10	(DE ‘ATTENTION‐deficit hyperactivity disorder’ OR DE ‘BEHAVIOR disorders’) OR (DE ‘SELF‐destructive behavior’ OR DE ‘SELF‐mutilation’ OR DE ‘SELF‐injurious behavior’)	4216
S9	(DE ‘EATING disorders’ OR DE ‘BINGE‐eating disorder’ OR DE ‘BULIMIA’ OR DE ‘COMPULSIVE eating’) OR (DE ‘ANOREXIA nervosa ‐‐ Social aspects’)	2397
S8	DE ‘DEPERSONALIZATION’	297
S7	(((((DE ‘PERSONALITY disorders’) OR (DE ‘ANTISOCIAL personality disorders’)) OR (DE ‘BORDERLINE personality disorder’)) OR (DE ‘NARCISSISTIC personality disorder’)) OR (DE ‘OBSESSIVE‐compulsive personality disorder’)) OR (DE ‘SCHIZOPHRENIA’)	6574
S6	DE ‘BIPOLAR disorder’	392
S5	DE ‘AUTISM spectrum disorders’	344
S4	((DE ‘AFFECTIVE disorders’) OR (DE ‘MENTAL depression’)) OR (DE ‘MENTAL illness ‐‐ Seasonal variations’)	10,872
S3	((DE ‘ANXIETY disorders’ OR DE ‘SOCIAL anxiety’) OR (DE ‘OBSESSIVE‐compulsive disorder’)) OR (DE ‘SOCIAL phobia’)	2345
S2	(DE ‘MENTAL illness’) OR (DE ‘PEOPLE with mental illness’)	11,110
S1	SU Mental disorder* OR mental disease* OR Mental illness* OR mental health OR Psychiatric diagnose OR Psychiatric illness* OR Psychiatric disease*OR Anxiety disorder* OR Phobic disorder* OR Social Anxiety OR ‘Generalized Anxiety Disorder’ OR ‘Obsessive Compulsive Disorder’ OR ‘Panic Attack’ OR ‘Panic Disorder’ OR ‘Acrophobia’ OR ‘Agoraphobia’ OR ‘Claustrophobia’ OR ‘Social Phobia’ OR OCD OR Antisocial disorder* OR Dissociative Disorder* OR Attention Deficit Disorder OR ADHD OR Hyperkinetic disorder* OR Asperger* OR Mood disorder* OR borderline OR Neurotic disorder* OR Personality disorder* OR Depression OR Bipolar disorder OR Schizophrenia OR Posttraumatic Stress Disorder OR Post‐traumatic Stress Disorder OR PTSD OR paranoia OR Psychosis OR Incest OR DE Pedophil* OR Voyeurism OR Eating Disorder* OR Anorexia OR Binge Eating OR Bulimia OR Narcissism OR Self‐Destructive Behavior OR Dissociative Disorder* OR Depersonalization OR Psychotic State OR borderline OR borderliner* OR neurosis OR Personality Disorder* OR Antisocial Personality Disorder OR Avoidant Personality Disorder OR Dependent Personality Disorder OR Narcissistic Personality Disorder OR Obsessive Compulsive Personality Disorder OR Paranoid Personality Disorder OR Passive Aggressive Personality Disorder OR Sadomasochistic Personality OR Schizoid Personality Disorder OR Schizophrenia OR bipolar disorder OR mania OR Autism OR autistic OR Affective Disorder* OR Disruptive Mood Dysregulation Disorder* OR mood disorder OR Major Depression OR Depression OR Endogenous Depression’ OR ‘Affective Psychosis’ OR ‘Schizoaffective Disorder’	157,254

#### 3.4.2 Searching other resources

##### 3.4.2.1 Searching other resources

Google Scholar—https://scholar.google.com
Google—https://www.google.com/

##### 3.4.2.2 Searches in Google and Google scholar will be performed with all meaningful combinations of key terms

Social Science Research Network—https://papers.ssrn.com/sol3/DisplayAbstractSearch.cfm
CORE—https://core.ac.uk Internationale repositorier
Danish National Research Database—http://www.forskningsdatabasen. dk/en
NORA—Norwegian Open Research Archives—http://nora. openaccess. no/
Cristin—Current Research Information SysTem In Norway—https://wo.cristin.no/as/WebObjects/cristin.woa/wa/fres?la=no
SwePub—Academic publications at Swedish universities—http://swepub.kb.se/
DIVA—https://www.diva-portal.org/smash/search.jsf?dswid=69

##### 3.4.2.3 Searches for working papers and conference proceedings in English

SHS Web of Conferences (www.shs-conferences.org) Open Access proceedings in Humanities and Social Sciences
The Social Care Institute for Excellence (SCIE): www.scie.org.uk/publications/index.asp

##### 3.4.2.4 Searches for Government Documents

NICE National Institute for Health and Care Excellence www.nice.org.uk

##### 3.4.2.5 Searches for Dissertations

EBSCO Open Dissertations (https://biblioboard.com/opendissertations/)
Open Access Theses and Dissertations (oatd.org)

##### 3.4.2.6 Hand Searches

The following Journals will be hand searched:

To find the relevant journals we used the following ‘journal suggesters’: Springer: https://journalsuggester.springer.com/; Elsevier: https://journalfinder.elsevier.com/and Health science overall: http://jane.biosemantics.org/. To guide the research in the journal suggesters, we used the title of the protocol and the following key words: group‐based community interventions, social reintegration, mental illness and marginalisation.

- BMC Public health
- BMC psychiatry
- Journal of Psychosocial Rehabilitation and Mental Health
- Psychiatric Quarterly
- Community Mental Health Journal
- Disability and rehabilitation
- International journal of mental health systems
- Sociology of health and illness

##### 3.4.2.7 Citation‐tracking

We will check the references for all identified existing systematic reviews and meta‐analyses and of all included primary studies.

##### 3.4.2.8 Contacting experts in the field

If during the search and screening process, we become aware of relevant experts in the field, these will be contacted and asked to provide information about relevant ongoing studies.

##### 3.4.2.9 Language restrictions

We will review studies published in English, Danish, Swedish and Norwegian.

### 3.5 Data collection and analysis

#### 3.5.1 Description of methods used in primary research

Based on the existing reviews we expect to be able to mostly include randomised trials.

An example of a study, which we will include is Eklund et al. (2017). This cluster‐randomised trial evaluated the effectiveness of a 16‐week group‐based intervention called Balancing Everyday Life (BEL) program, compared to care as usual (CAU) for people with mental illness in specialised (out‐patient) and community‐based psychiatric services. BEL is a group‐based program (5–8 participants) consisting of 12 sessions, 1 session a week, and 2 booster sessions with 2‐week intervals. The themes for the group sessions are, for example, activity balance, meaning and motivation, healthy living, work‐related activities, leisure and relaxation, and social activities. Each session contains a brief educational section, a main group activity and a home assignment to be completed between sessions. The main group activity starts with analysing the past and (foremost) the present situation and proceeds with identifying desired activity goals and finding strategies for how to reach them. This mapping and planning step is followed by a home assignment that means performing the desired activity in a real‐life context. The home assignment is aimed at testing one of the proposed strategies. During the next group meeting, the real‐life experience is evaluated and group members discuss and give each other feedback. Goals and strategies may be re‐negotiated, if needed. The main outcomes of the trial included different aspects of subjectively evaluated everyday activities, in terms of the engagement and satisfaction they bring, balance among activities, and activity level. Secondary outcomes included various facets of well‐being and functioning. The BEL group included 133 participants and the CAU group 93. They completed self‐report questionnaires targeting activity and well‐being on three occasions—at baseline, after completed intervention (at 16 weeks) and at a 6‐month follow‐up. A research assistant rated the participants' level of functioning and symptom severity on the same occasions.

#### 3.5.2 Criteria for determination of independent findings

To determine the independence of results in included studies, we will consider whether individuals may have undergone multiple interventions, whether there were multiple treatment groups and whether several studies are based on the same data source as well as whether studies yield results from multiple eligible sample populations. The first three scenarios create correlation among error terms of the effect sizes, whereas the latter scenario produces dependence among the mean effects from a given study. For a more comprehensive description of the analysis strategy see the *Data synthesis* section.

##### 3.5.2.1 Multiple interventions groups and multiple interventions per individual

Studies with multiple intervention groups with different individuals will be included in this review, although only intervention and control groups that meet the eligibility criteria will be used in the data synthesis. Results from studies that either apply multiple eligible intervention or control groups will be correlated since they are based on overlapping samples. This creates what is called *a correlated effects dependency structure* among effect sizes. To avoid problems with dependence between effect sizes we will apply Robust Standard Errors (RVE; Hedges et al., 2010; Pustejovsky & Tipton, 2021) and use the small sample adjustment to the estimator itself (Tipton, 2015; Tipton & Pustejovsky, 2015). We apply the newly‐developed correlated‐hierarchical effects (CHE) models that guard against any model mis‐specification via RVE since these models (CHE‐RVE) imply that we can account for various types of dependencies among effect sizes (Pustejovsky & Tipton, 2021). Furthermore, this method has shown to be the most accurate to handle dependent effect sizes (Fernández‐Castilla, Aloe, et al., 2020; Vembye Mikkel et al., 2022). See Section Data Synthesis below for more details about the data synthesis. We will use the degrees of freedom from all RVE models as diagnostics for the certainty in our variance estimation to either evaluate the impact of the number of studies or the balance of the covariates (Tipton, 2015; Tipton & Pustejovsky, 2015; Pustejovsky & Tipton, 2021).

We do not apply aggregated effect sizes since it has been shown that this technique does not control the nominal Type I error rate, i.e., it yields too many false‐positive results (Moeyaert et al., 2017; Vembye Mikkel et al., 2022), when dependencies among effect sizes are widespread in the meta‐analytical data, as we expect to find.

##### 3.5.2.2 Multiple studies using the same sample of data

In some cases, several studies may have used the same sample of data or some studies may have used only a subset of a sample used in another study. We will review all such studies, but in the meta‐analysis we will only include one estimate of the effect from each sample of data. This will be done to avoid dependencies between the ‘observations’ (i.e., the estimates of the effect) in the meta‐analysis. The choice of which estimate to include will be based on our risk of bias assessment of the studies. We will choose the estimate from the study that we judge to have the least risk of bias (primarily, Confounding bias). If two (or more) studies are judged to have the same risk of bias and one of the studies (or more) uses a subset of a sample used in another study (or studies) we will include the study using the full set of participants.

##### 3.5.2.3 Multiple time points

When the results are measured at multiple time points, we plan to model time differences via appropriate CHE models so that we can reliably estimate and compare confidence intervals and mean differences among time points (Pustejovsky & Tipton, 2021; Tipton & Pustejovsky 2015). As a general guideline, these will be grouped together as follows: (1) postintervention, that is, less than a year follow‐up, (2) 1–2‐year follow up, and (3) More than 2 year follow up. However, should the studies provide viable reasons for an adjusted choice of relevant and meaningful duration intervals for the analysis of outcomes, we will adjust the grouping.

##### 3.5.2.4 Multiple samples within the same study

It might happen that some studies report results across multiple nonoverlapping samples. Although the effect sizes come from independent samples the fact that authors used the same sampling, estimation techniques, etc., creates dependence among the mean effects from studies also known as *hierarchical effects dependency structure*. Our need for the opportunity to both account for correlated as well as hierarchical effects dependency structures emphasizes why we apply the new RVE‐methods (Pustejovsky & Tipton, 2021).

#### 3.5.3 Selection of studies

Under the supervision of review authors, two review team assistants will first independently screen titles and abstracts to exclude studies that are clearly irrelevant. Studies considered eligible by at least one assistant or studies were there is insufficient information in the title and abstract to judge eligibility, will be retrieved in full text. The full texts will then be screened independently by two review team assistants under the supervision of the review authors. Any disagreement of eligibility will be resolved by the review authors. Exclusion reasons for studies that otherwise might be expected to be eligible will be documented and presented in an appendix.

The study inclusion criteria will be piloted by the review authors (see Appendix *First and second level screening*). The overall search and screening process will be illustrated in a flow diagram. None of the review authors will be blind to the authors, institutions, or the journals responsible for the publication of the articles.

#### 3.5.4 Data extraction and management

Two review authors will independently code and extract data from included studies. A coding sheet will be piloted on several studies and revised as necessary (see Appendix *Data extraction*). Disagreements will be resolved by consulting a third review author with extensive content and methods expertise. Disagreements resolved by a third reviewer will be reported. Data and information will be extracted on: available characteristics of participants, intervention characteristics and control conditions, research design, sample size, risk of bias and potential confounding factors, outcomes, and results. Extracted data will be stored electronically. Analysis will be conducted using RevMan5 and Stata software.

#### 3.5.5 Assessment of risk of bias in included studies

We will assess the risk of bias in randomised studies using Cochranes revised risk of bias tool, ROB 2 (Higgins et al., 2019).

The tool is structured into five domains, each with a set of signalling questions to be answered for a specific outcome. The five domains cover all types of bias that can affect results of randomised trials.

The five domains for individually randomised trials are:

1. bias arising from the randomisation process;
2. bias due to deviations from intended interventions (separate signalling questions for effect of assignment and adhering to intervention);
3. bias due to missing outcome data;
4. bias in measurement of the outcome;
5. bias in selection of the reported result.

For cluster‐randomised trials, an additional domain is included ((1b) Bias arising from identification or recruitment of individual participants within clusters). We will use the latest template for completion (currently it is the version of 15 March 2019 for individually randomised parallel‐group trials and 2021 Marts for cluster‐randomised trials). In the cluster randomised (CRCT) template (Eldridge et al., 2021), however, only the risk of bias due to deviation from the intended intervention (effect of assignment to intervention; intention to treat ITT) is present and the signalling question concerning the appropriateness of the analysis used to estimate the effect is missing. Therefore, for cluster randomised trials we will only use the signalling questions concerning the bias arising from identification or recruitment of individual participants within clusters from the template for cluster randomised parallel‐group trials; otherwise, we will use the template and signalling questions for individually randomised parallel‐group trials.

We will assess the risk of bias in non‐randomised studies, using the model ROBINS –I, developed by members of the Cochrane Bias Methods Group and the Cochrane Non‐Randomised Studies Methods Group (Sterne et al., 2016a). We will use the latest template for completion (currently it is the version of September 19, 2016).

The ROBINS‐I tool is based on the Cochrane RoB tool for randomised trials, which was launched in 2008 and modified in 2011 (Higgins et al, 2011).

The ROBINS‐I tool covers seven domains (each with a set of signalling questions to be answered for a specific outcome) through which bias might be introduced into nonrandomised studies:

1. bias due to confounding;
2. bias in selection of participants;
3. bias in classification of interventions;
4. bias due to deviations from intended interventions;
5. bias due to missing outcome data;
6. bias in measurement of the outcome; and
7. bias in selection of the reported result.

The first two domains address issues before the start of the interventions and the third domain addresses classification of the interventions themselves. The last four domains address issues after the start of interventions and there is substantial overlap for these four domains between bias in randomised studies and bias in non‐randomised studies trials (although signalling questions are somewhat different in several places, see Sterne et al., 2016b and Higgins et al., 2019).

Randomised study outcomes are rated on a ‘Low/Some concerns/High’ scale on each domain; whereas non‐randomised study outcomes are rated on a ‘Low/Moderate/Serious/Critical/No Information’ scale on each domain. The level ‘Critical’ means: the study (outcome) is too problematic in this domain to provide any useful evidence on the effects of intervention and it is excluded from the data synthesis. The same critical level of risk of bias (excluding the result from the data synthesis) is not directly present in the RoB 2 tool, according to the guidance to the tool (Higgins et al., 2019).

We will add a critical level of risk of bias to the RoB 2 tool with the same meaning as in the ROBINS‐I tool; that is, the study (outcome) is too problematic in this domain to provide any useful evidence on the effects of intervention and it is excluded from the data synthesis. We will stop the assessment of a randomised study outcome using the RoB 2 as soon as one domain is judged as ‘Critical’. Likewise, we will stop the assessment of a non‐randomised study outcome as soon as one domain in the ROBINS‐I is judged as ‘Critical’.

‘High’ risk of bias in multiple domains in the RoB 2 assessment tool may lead to a decision of an overall judgement of ‘Critical’ risk of bias for that outcome and it will be excluded from the data synthesis. ‘Serious’ risk of bias in multiple domains in the ROBINS‐I assessment tool may lead to a decision of an overall judgement of ‘Critical’ risk of bias for that outcome and it will be excluded from the data synthesis.

##### 3.5.5.1 Confounding

An important part of the risk of bias assessment of non‐randomised studies is consideration of how the studies deal with confounding factors. Systematic baseline differences between groups can compromise comparability between groups. Baseline differences can be observable (e.g., age and gender) and unobservable (to the researcher; e.g., motivation and ‘ability’). There is no single non‐randomised study design that always solves the selection problem. Different designs represent different approaches to dealing with selection problems under different assumptions, and consequently require different types of data. There can be particularly great variations in how different designs deal with selection on unobservables. The ‘adequate’ method depends on the model generating participation, that is, assumptions about the nature of the process by which participants are selected into a programme.

As there is no universal correct way to construct counterfactuals for non‐randomised designs, we will look for evidence that identification is achieved, and that the authors of the primary studies justify their choice of method in a convincing manner by discussing the assumption(s) leading to identification (the assumption(s) that make it possible to identify the counterfactual). Preferably the authors should make an effort to justify their choice of method and convince the reader that the only difference between a treated individual and a nontreated individual is the treatment. The judgement is reflected in the assessment of the confounder unobservables in the list of confounders considered important at the outset (see Supporting Information: Appendix *User guide for unobservables*).

In addition to unobservables, we have identified the following observable confounding factors to be most relevant: age, gender and risk indicators as described in section *Type of participants*. In each study, we will assess whether these factors have been considered, and in addition we will assess other factors likely to be a source of confounding within the individual included studies. If studies do not ensure baseline equivalence among intervention groups, they either have to provide pretest or baseline measures from which we can calculate pretest‐/baseline‐adjusted effect sizes, otherwise nonequivalent group designed studies will be excluded due to a critical risk of confounding.

##### 3.5.5.2 Importance of pre‐specified confounding factors

The motivation for focusing on age, gender, and risk indicators is given below.

The prevalence of different types of behavioural and psychological problems, coping skills, cognitive and emotional abilities vary throughout human development through puberty and into adulthood, and therefore we consider age to be a potential confounding factor. Furthermore, there are substantial gender differences in behaviour problems, coping and risk of different types of adverse outcomes which is why we also include gender as a potential confounding factor (Card et al., 2008; Hampel & Petermann, 2005).

Pretreatment group equivalence on mental illness such as primary diagnosis and comorbid conditions/problems such as alcohol/substance use, homelessness, poverty, etc., are indisputable important confounders as the magnitude and severity of pre‐existing conditions and problems within the target population is very likely to be associated with treatment effects (Compton et al., 2003). Therefore, the accuracy of the estimated effects of group‐based interventions will likely depend crucially on how well these factors are controlled for.

##### 3.5.5.3 Effect of primary interest and important co‐interventions

We are mainly interested in the effect of starting and adhering to the intended intervention, that is, the treatment on the treated (TOT) effect. The risk of bias assessments will therefore be in relation to this specific effect. Important co‐interventions may include psychopharmacological treatment or other active treatments such as individual psychotherapy, mentoring or counselling.

#### 3.5.6 Measures of treatment effect

##### 3.5.6.1 Continuous outcomes

For continuous outcomes, effects sizes with 95% confidence intervals will be calculated, where means and standard deviations are available. If means and standard deviations are not available, we will calculate SMDs from various sources tailored to the given research design and estimation technique as sugged by Lipsey and Wilson (2001) and others (Pustejovsky, 2016; WWC, 2020, 2021). If not enough information is yielded, the review authors will request this information from the principal investigators. Hedges' *g* will be the estimator (Hedges, 1981) used for estimating standardised mean differences (SMD). Any measures of drug and alcohol use or social and emotional outcomes, are examples of relevant continuous outcomes in this review.

##### 3.5.6.2 Dichotomous outcomes

For dichotomous outcomes, we will calculate odds ratios with 95% confidence intervals. Hospital readmission, drop‐out, criminal behaviour and homelessness, are examples of relevant dichotomous outcomes in this review.

There are statistical approaches available to re‐express dichotomous and continuous data to be pooled together (Sánchez‐Meca et al., 2003). To calculate common metric odds ratios will be converted to SMD effect sizes using the Cox transformation. We will only transform dichotomous effect sizes to SMD if appropriate, for example, as may be the case with for example the outcomes drug and alcohol use, that can be measured with binary and continuous data.

When effect sizes cannot be pooled, study‐level effects will be reported in as much detail as possible. Software for storing data and statistical analyses will be RevMan Web, Excel, R, and Stata 17.0.

#### 3.5.7 Unit of analysis issues

Errors in statistical analysis can occur when the unit of allocation differs from the unit of analysis. In cluster randomised trials, participants are randomised to treatment and control groups in clusters, either when data from multiple participants in a setting are included (creating a cluster within the community setting), or when participants are randomised by treatment locality. Non‐randomised studies may also include clustered assignment of treatment. Effect sizes and standard errors from such studies may be biased if the unit‐of‐analysis is the individual and an appropriate cluster adjustment is not used (Higgins & Green, 2011).

If possible, we will adjust effect sizes individually using the methods suggested by Hedges (2007) and information about the intra‐cluster correlation coefficient (ICC), realised cluster sizes, and/or estimates of the within and between variances of clusters. If it is not possible to obtain this information, we will adjust effect sizes using estimates from the literature (we will search for estimates of relevant ICC's), and assume equal cluster sizes. To calculate an average cluster size, we will divide the total sample size in a study by the number of clusters.

#### 3.5.8 Dealing with missing data

Missing data and attrition rates will be assessed in the included studies; see section *Assessment of risk of bias in included studies*. Where studies have missing summary data, such as missing standard deviations, the review authors will request this information from the principal investigators. If no information is yielded, we will calculate SMDs from various sources tailored to the given research design and estimation technique as sugged by Lipsey & Wilson (2001) and others (Pustejovsky, 2016; WWC, 2020, 2021). If missing summary data cannot be derived, the study results will be reported in as much detail as possible.

#### 3.5.9 Assessment of heterogeneity

Heterogeneity among primary outcome studies will be assessed with *χ* ^2^ (*Q*) test, and the *I* ^2^, and *τ* ^2^ (between‐study/study‐level variation—expressed as SD) (Higgins et al., 2003), and *ω* ^2^ (within‐study/effect size level variation—expressed as SD) (Pustejovsky & Tipton, 2021; Van den Noortgate et al., 2013). If further levels of variation appear to be present in our data, we will add this/these to our models. Any interpretation of the *χ* ^2^ test will be made cautiously on account of its low statistical power.

#### 3.5.10 Assessment of reporting biases

Reporting bias refers to both publication bias and selective reporting of outcome data and results. Here, we state how we will assess publication bias.

We will use funnel plots tailored for analysis of dependent effect sizes (Fernández‐Castilla, Declercq, et al., 2020) for information about possible publication bias if we find sufficient studies (Higgins & Green, 2011; Pustejovsky & Rodgers, 2019; Rodgers & Pustejovsky, 2021). However, asymmetric funnel plots are not necessarily caused by publication bias (and publication bias does not necessarily cause asymmetry in a funnel plot). If asymmetry is present, we will consider possible reasons for this.

#### 3.5.11 Data synthesis

The overall data synthesis will be conducted where effect sizes are available or can be calculated, and where studies are similar in terms of the outcome measured. Meta‐analysis of outcomes will be conducted on each metric (as outlined in section *Types of outcomes measures*) separately.

Since studies might report different outcomes for the same construct of measurement, for example, varying between binary and continuous constructs, which in turn may produce effect sizes that as such are not comparable, we will be transparent about all methods used in the primary studies (research design and statistical analysis strategies) and use caution when synthesising effect sizes that come from different construct scales. We do not intend to amalgamate results across any of the outcome categories mentioned in the Types of outcome measures section.

When the effect sizes used in the data synthesis are odds ratios, they will be log transformed before being analysed. The reason is that ratio summary statistics all have the common feature that the lowest value that they can take is 0, that the value 1 corresponds with no intervention effect, and the highest value that an odds ratio can ever take is infinity. This number scale is not symmetric. The log transformation makes the scale symmetric: the log of 0 is minus infinity, the log of 1 is zero, and the log of infinity is infinity.

Studies that have been coded with a Critical risk of bias will not be included in the data synthesis.

As the intervention deal with diverse populations of participants and we, therefore, expect heterogeneity among primary study outcomes, all analyses of the overall effect will be inverse variance (under the assumed working model) weighted using random effects CHE‐RVE models (Pustejovsky & Tipton, 2021; Vembye et al., 2022) that incorporate both the sampling variance (*σ* ^2^), the assumed sample correlation (*ρ*), as well as the within‐ (*ω* ^2^) and between‐study (*τ* ^2^) variance components into the study level weights (Pustejovsky, 2020; Viechtbauer, 2021). Random effects weighted mean effect sizes will be calculated using 95% confidence intervals and we will provide a graphical display (forest plot) of effect sizes (Fernández‐Castilla, Declercq, et al., 2020). Graphical displays for meta‐analysis performed on ratio scales sometimes use a log scale, as the confidence intervals then appear symmetric. We will use R to generate these plots.^1^ Heterogeneity among primary outcome studies will be assessed with *χ* ^2^ (*Q*) test, and the *I* ^2^, and *τ* ^2^ (between‐study/study‐level variation—expressed as SD) (Higgins Julian et al., 2003), and *ω* ^2^ (within‐study/effect size level variation—expressed as SD) (Pustejovsky & Tipton, 2021; Van den Noortgate et al., 2013). If further levels of variation appear to be present in our data, we will add this/these to our models. Any interpretation of the *χ* ^2^ test will be made cautiously on account of its low statistical power.

For subsequent analyses of moderator variables that may contribute to systematic variations, we will either use the CHE model, the subgroup correlated effects (SCE) mode, or the correlated multivariate effects (CMVE) models, depending on data structure of the meta‐regression test, and we will use Cluster Wild Bootstrapping techniques to estimate *p* values since these have shown to be the most accurate and powerful approach to obtaining *p* values for meta‐regression (Joshi et al., 2022). We correct for multiplicity by using the false discovery rate (FDR) method sugged by Polanin (2013).

Several studies may have used the same sample of data. We will review all such studies, but in the meta‐analysis we will only include one estimate of the effect from each sample of data. This will be done to avoid dependencies between the ‘observations’ (i.e., the estimates of the effect) in the meta‐analysis. The choice of which estimate to include will be based on our quality assessment of the studies. We will choose the estimate from the study that we judge to have the least risk of bias, with particular attention paid to Confounding bias.

Studies may provide results separated by for example age and/or gender. We will include results for all age and gender groups. To take into account the dependence between such multiple effect sizes from the same study, we will apply correlated‐hierarchical effects models that both take into account the multi‐level structure of the data (with effect sizes nested in samples that are nested in studies) and the correlation among effect sizes while guarding against any mis‐specifications via RVE (Hedges et al., 2010; Pustejovsky & Tipton, 2021). An important feature of this analysis is that the results are valid regardless of the weights used. When the models are correctly specified the used weights will be fully efficient. Using restricted maximum likelihood techniques (Viechtbauer, 2005), we will estimate two sources of heterogeneity, that is, the standard deviations at the effect size level (also known as the within‐study SD, *ω*) and at the study level (also known as the between‐study SD, *τ*). We will assume that effect sizes are equicorrelated. The assumed correlation is a rough approximation given that *ρ* is, in fact, unknown and the correlation structure may be more complex. We will calculate weights using estimates of *τ* ^2^, *ω* ^2^, and overall SD by setting *ρ* = 0.80 and conduct sensitivity tests using a variety of *ρ* values; to asses if the general results and estimates of the heterogeneity are robust to the choice of *ρ*. For all tests, we will use the CR2 small sample adjustment as proposed by Bell and McCaffrey (2002) and extended by McCaffrey et al. (2001) and in meta‐analysis extended by Tipton (2015), Pustejovsky and Tipton (2015, 2021), and Joshi et al. (2022) together with Satterthwaite degrees of freedom (Satterthwaite, 1946). We will use the degrees of freedom from all RVE models as diagnostics for the certainty in our variance estimation to either evaluate the impact of the number of studies or the balance of the covariates (Pustejovsky & Tipton, 2021; Tipton, 2015; Tipton & Pustejovsky, 2015).

#### 3.5.12 Subgroup analysis and investigation of heterogeneity

We will investigate the following factors with the aim of explaining potential observed heterogeneity: participant's psychiatric diagnoses, age and gender of participants, type of intervention (primary aim of intervention, duration, and intensity of intervention), and theoretical perspective informing the intervention (e.g., CBT, social skills, etc.).

If the number of included studies is sufficient and given there is variation in the covariates (age, gender, diagnoses, and type of intervention), we will perform moderator analyses (multiple meta‐regression using the CHE‐RVE models) to explore how observed variables are related to heterogeneity.

If there are a sufficient number of studies, we will apply the CHE‐RVE working model family with inverse variance weights (given that our working models are correctly specified) calculated using a method proposed by Pustejovsky and Tipton (2021). This technique calculates standard errors using an empirical estimate of the variance: it does not require any assumptions regarding the distribution of the effect size estimates. The assumptions that are required to meet the regularity conditions are minimal and generally met in practice. For categorical moderator variables, we will either use the Subgroup Correlated Effects model or the Correlated Multivariate Effects (CMVE) model. The main difference between the SCE and CMVE models is that the CMVE model both allows effect sizes coming from the same studies that fall into the same subgroup but also effect sizes that fall into different subgroups to be correlated, whereas the SCE model only allows correlation among effect sizes from the same study failing in the same subgroup but not effect sizes from the same study falling into different subgroup dimensions (see Pustejovsky & Tipton, first preprint version). Although the CMVE model is based on more realistic assumptions and is superior relative to the SCE in terms of precision, the CMVE model only works under narrow conditions when (1) there are few multivariate dimensions, (2) there are a substantial number of studies and effect sizes available in each dimension, and (3) there are a substantial number of studies having effect sizes from each possible pair of outcome dimensions. Whenever these conditions are met, we use the CMVE model. However, since these conditions are rather restricted, we expect that the SCE model will be the main working horse for our meta‐regression analyses. If large amount of the within‐study heterogeneity remains across subgroups, we will add this level of variance to the models as suggested by Pustejovsky and Tipton (2021). For continuous moderator variables, we will apply the same CHE‐RVE working model as for the overall mean effect size estimation. For all models, we assume *ρ* = 0.8 and conduct sensitivity tests using a variety of *ρ* values; to assess if the general results including variance estimation are robust to the choice of *ρ*. Furthermore, for all models, we apply the same sample adjustment technique and Satterthwaite degrees of freedom as for the overall mean effect size estimation. Also, we will use the degrees of freedom from all RVE models as diagnostics for the certainty in our variance estimation to either evaluate the impact of the number of studies or the balance of the covariates (Pustejovsky & Tipton, 2021; Tipton, 2015; Tipton & Pustejovsky, 2015). We will estimate the correlations between the covariates and consider the possibility of confounding. Conclusions from meta‐regression analysis will be cautiously drawn and will not solely be based on significance tests since the power for meta‐regression models is generally low. The magnitude of the coefficients and width of the confidence intervals will be taken into account as well. We will use Wald Tests with Cluster Wild Bootstrapping to contrast differences among subgroup categories (Joshi et al., 2022). Interpretation of relationships will be cautious, as they are based on a subdivision of studies and indirect comparisons. Although our meta‐regression results cannot firmly clinch causality, we will interpret our meta‐regression analyses as indications of causal signs relevant for future primary research and investigation (Cook et al., 1992).

In general, the strength of inference regarding differences in treatment effects among subgroups is controversial when based on variables that entail within‐study variation since between‐study differences can entail a higher risk of indicating relations at the aggregate level that does not hold at the study level; see Oxman and Guyatt (1992). We will therefore use within‐study differences where possible, i.e., compare effect sizes based on male or female samples instead of, for example, using the aggregate measure of the percent of females in the sample.

We will also consider the degree of consistence of differences, as making inferences about different effect sizes among subgroups entails a higher risk when the difference is not consistent within the studies; see Oxman and Guyatt (1992).

#### 3.5.13 Sensitivity analysis

Sensitivity analysis will be carried out by restricting the meta‐analysis to a subset of all studies included in the original meta‐analysis and will be used to evaluate whether the pooled effect sizes are robust across components of risk of bias. We will consider sensitivity analysis for each domain of the risk of bias checklists and restrict the analysis to studies with a low risk of bias. Also, we will conduct leave‐one‐study‐out sensitivity analyses to investigate the impact of each study on the effect size estimations.

Sensitivity analyses with regard to research design and statistical analysis strategies in the primary studies will be an important element of the analysis to ensure that different methods produce consistent results.

##### 3.5.13.1 Treatment of qualitative research

We do not plan to include qualitative research.

#### 3.5.14 Summary of findings and assessment of the certainty of the evidence

In the full review, we will provide summary of findings tables and an assessment of the certainty of the evidence based on the included studies.

## CONTRIBUTIONS OF AUTHORS

Nina T. Dalgaard is a psychologist, Ph.D. Nina has content knowledge about mental illness and social marginalisation as well as knowledge about systematic review methods.

Karl F. Krassel, economist, Ph.D. with knowledge of program evaluation and statistical methods. Karl Fritjof is project manager of the proposed review and will be involved in all steps throughout the review. Karl Fritjof will also be responsible of the statistical analysis.

Maya C. Flensborg Jensen, Ph.D. Maya has content knowledge with respect to community interventions, social marginalisation and mental illness. Maya will contribute designing the review and participate in reviewing the found literature.

Elizabeth Bengtsen (information specialist) is an experienced research librarian and search specialist, who has worked for core research institutions in Denmark for many years. Elizabeth has acquired expertise in database searching within various fields of research and in the context of systematic review searching.

Mikkel H. Vembye, Associate Methods Editor, Social Welfare, Campbell Collaboration. Mikkel is an expert in statistical inference in meta‐analysis involving dependent effect sizes.

## DECLARATIONS OF INTEREST

Please declare any potential conflicts of interest. For example, have any of the authors been involved in the development of relevant interventions, primary research, or prior published reviews on the topic?

## Supporting information

Supporting information.

## Footnotes

^1^
If we apply robust variance estimation, the analysis will be conducted in R.
