# Supplementary material for: PROTOCOL: Group‐based community interventions to support the social reintegration of marginalised adults with mental illness
Source: Campbell Syst Rev. 2022 Jul 14;18(3):e1254. doi: 10.1002/cl2.1254 (PMC9283853; doi:10.1002/cl2.1254)
Supplement: Supplementary file 1 — Supporting information. [file CL2-18-e1254-s001.docx]

# Appendices

## 1 First and second level screening

## First level screening is on the basis of titles and abstracts. Second level is on the basis of full text

Reference id. No. :

Reviewers initials:

Source:

Year of publication:

Country/countries of origin:

Author(s):

The study will be excluded if one or more of the answers to question 1-4 are ‘No’. If the answers to question 1 to 4 are ‘Yes’ or ‘Uncertain’, then the full text of the study will be retrieved for second level eligibility. All unanswered questions need to be posed again on the basis of the full text. If not enough information is available, or if the study is unclear, the author of the study will be contacted if possible.

**Screening questions:**

1. Does the study focus on an intervention offered to adults suffering from mental illness?

Yes - include

No – if no then stop here and exclude

Uncertain - include

Question 1 guidance:

The population of this review are adults suffering from any kind of mental illness. Studies focusing on interventions for at risk adults more broadly will not be eligible.

1. Is the intervention delivered in a group format?

Yes - include

No – if no then stop here and exclude

Uncertain – include

Question 2 guidance:

The intervention must be delivered in a group format mening that more than one participant receive the intervention at the same time and place.

1. Is the intervention aimed at more than symptom reduction? (this may be difficult to determine based on the title and abstract alone)

Yes - include

No – if no then stop here and exclude

Uncertain – include

Question 3 guidance:

In order to be eligible for the review, the aims of the intervention and outcomes for the study must be broader tan simply to reduce symptoms of a specific illness. Thus included interventions should also targets one or more of the following personal/social problems:

- Alcohol/substance abuse,
- Self-harming behaviour,
- Criminal behaviour,
- Homelessness,
- Poverty,
- Unemployment,
- Hospital admissions,
- Participants’ subjective well-being and quality of life

1. Is the report/article a quantitative evaluation study with a comparison condition? If not, does the paper make reference to a normative sample for comparison of attachment distributions pre- and post intervention?

Yes - include

No – if no then stop here and exclude

Uncertain – include

Question 4 guidance:

We are only interested in primary quantitative studies with a comparison group. We are not interested in theoretical papers on the topic or surveys/reviews of studies of the topic. (This question may be difficult to answer on the base of titles and abstracts alone.).

## 2 Data Extraction

| **Names of author(s)** |
| --- |
| **Title** |
| **Language** |
| **Journal** |
| **Year** |
| **Country** |
| **Participant characteristic (age, gender, type of mental illness)** |
| **Programme feature** *Format, Therapeutic perspective and technique* |
| **Programme feature** *Treatment goals/aims for the intervention* |
| **Programme feature** *Therapist characteristics, educational background, years of experience* |
| **Programme feature** *Duration* **(**total duration, number of sessions and duration of sessions**)** |
| **Programme feature** *Setting (outpatient clinic, community facility)* |
| **Type of data used in study (independent observation, questionnaire, other (specify))** |
| **Level of aggregation (individual and/or group based)** |
| **Time period covered by analysis (divide into intervention and follow up)** |
| **Sample size (divide into treated/comparison)** |

**Outcome measures**

Instructions: Please enter outcome measures in the order in which they are described in the report. Note that a single outcome measure can be completed by multiple sources and at multiple points in time (data from specific sources and time-points will be entered later).

| # | Outcome  & measure | Reliability & Validity | Format | Direction | Pg# & notes |
| --- | --- | --- | --- | --- | --- |
| 1 |  | Info from:  Other samples  This sample  Unclear  Info provided: | Dichotomy  Continuous  Categorical | High score or event is  Positive  Negative  Can’t tell |  |

* Repeat as needed

**OUT COME DATA**

**DICHOTOMOUS OUTCOME DATA**

| OUTCOME | TIME POINT (s) (record exact time from participation, there may be more than one, record them all) | SOURCE | VALID Ns | CASES | NON-CASES | STATISTICS | Pg. # & NOTES |
| --- | --- | --- | --- | --- | --- | --- | --- |
|  |  | Questionnaire  Admin data  Other (specify)  Unclear | Participation | Participation | Participation | RR (risk ratio)  OR (odds ratio)  SE (standard error)  95% CI  DF  P- value (enter exact p value if available)  Chi2  Other |  |
|  |  |  |  |  |  |  |  |
|  |  |  | Comparison | Comparison | Comparison |  |  |
|  |  |  |  |  |  |  |  |

Repeat as needed
